# Novel rat model of gaming disorder: assessment of social reward and sex differences in behavior and c-Fos brain activity

**DOI:** 10.1007/s00213-024-06576-y

**Published:** 2024-04-05

**Authors:** Antonino Casile, Marilena Marraudino, Brigitta Bonaldo, Maria Vittoria Micioni Di Bonaventura, Sofia Nasini, Carlo Cifani, Stefano Gotti

**Affiliations:** 1 School of Pharmacy, Pharmacology Unit, Pharmacology Unit, University of Camerino, Via Madonna delle Carceri, 9, Camerino, 62032 Italy; 2 Neuroscience Institute Cavalieri Ottolenghi (NICO), University of Turin, Regione Gonzole, 10, Orbassano, Turin, TO 10043 Italy; 3 Department of Neuroscience “Rita Levi-Montalcini”, Via Cherasco 15, Turin, TO 10126 Italy; 4 Department of Health Sciences and Research Center on Autoimmune and Allergic Diseases (CAAD), University of Piemonte Orientale (UPO), Novara, Italy; 5 Laboratory of Molecular and Cellular Pharmacology, Department of Pharmacology, University of Padua, Largo Egidio Meneghetti, 2, Padua, 35131 Italy

**Keywords:** Gaming disorder (GD), Mental disorder, Sexual difference, Animal model, Social reward, Loss control, Hyperactivity, c-Fos

## Abstract

**Rationale:**

In 2018, the International Classification of Diseases (ICD-11) classified Gaming Disorder (GD) as a mental disorder. GD mainly occurs among adolescents, who, after developing addiction, show psychopathological traits, such as social anxiety, depression, social isolation, and attention deficit. However, the different studies conducted in humans so far show several limitations, such as the lack of demographic heterogeneity and equal representation of age, differences in the type of game and in the follow-up period. Furthermore, at present, no animal models specific to GD are available.

**Objectives:**

To address the lack of an experimental model for GD, in the present work, we proposed a new GD rat model to investigate some peculiar tracts of the disorder.

**Methods:**

Two-month-old Wistar Kyoto rats, both males and females, were subject to a five-week training with a new innovative touch-screen platform. After five weeks of training, rats were assessed for: (a) their attachment to the play under several conditions, (b) their hyperactivity during gaming, and (c) the maintenance of these conditions after a period of game pause and reward interruption. After sacrifice, using immunohistochemistry techniques, the immunoreactivity of c-Fos (a marker of neuronal activity) was analyzed to study different neural areas.

**Results:**

After the training, the rats subjected to GD protocol developed GD-related traits (e.g., hyperactivity, loss control), and the behavioral phenotype was maintained consistently over time. These aspects were completely absent in the control groups. Lastly, the analysis of c-Fos immunoreactivity in prelimbic cortex (PrL), orbitofrontal cortex (OFC), nucleus Accumbens, amygdala and bed nucleus of stria terminalis (BNST) highlighted significant alterations in the GD groups compared to controls, suggesting modifications in neural activity related to the development of the GD phenotype.

**Conclusions:**

The proposal of a new GD rat model could represent an innovative tool to investigate, in both sexes, the behavioral and neurobiological features of this disorder, the possible role of external factors in the predisposition and susceptibility and the development of new pharmacological therapies.

**Supplementary Information:**

The online version contains supplementary material available at 10.1007/s00213-024-06576-y.

## Introduction

Play is an important part of developing human behavior and consolidating new experiences (Paulus et al. 2018). Over the past 20 years, recreational activities have changed since the increased availability and use of computer technology have greatly raised (Paulus et al. 2018). Computer games, internet use, and social media have become common activities for children and adolescents (Paulus et al. 2018) engaging experience and producing a sense of accomplishment as they acquire new skills (Reynaldo et al. 2021), underestimating the negative aspects, probably due to the influence of “social desirability” (Jo et al. 2019). Recent studies showed that children and adolescents spend more than 8 to 10 h per day using various electronic media, such as television, computers, smartphones, and social media (Nobre et al. 2020). This phenomenon was amplified even more in 2020, right in the first phase of COVID-19 (Wijman 2020), when the number of players globally reached 2.6 billion people and video game sales were record-breaking (Schreier 2020; Porter 2021).

However, the term ‘gamers’ include individuals with various intra- and inter-personal risk factors who use computer games as a coping strategy for various issues (Kuss et al. 2014). Excessive use becomes a negative factor that limits real-life experiences (Kuss et al. 2014), so much so that it assumes a solid resemblance to addictive disorders. Therefore, “Gaming Disorder” (GD) was included in the 11th final revision of the International Classification of Diseases (ICD-11) by the World Health Organization (WHO) (American Psychiatric Association 2013) and recently (on May 25th, 2019) recognized as a medical disorder (WHO 2018).

GD is characterized by a repetitive or persistent gaming behavior pattern over a period of at least 12 months (WHO 2018). Specifically, the diagnosis of GD involves three symptoms: *(i)* impaired control over the game, *(ii)* increasing priority given to the game, and *(iii)* continuation or escalation of the game despite the occurrence of negative consequences (WHO 2018). Although GD is a clinically identifiable disorder, today it is not easy to understand the risk of developing it due to the lack of clear predictive signals and many potential contributing factors.

Interestingly, GD shows a higher prevalence in males compared to females (Mihara and Higuchi 2017). Furthermore, boys have greater gaming addiction during adolescence, when the time spent playing video games increases, while girls develop greater use of the game at an older age, when they generate an addiction to social networks (Lopez-Fernandez et al. 2019). Currently, the studies available on GD present a strong limitation in the sampling of subjects, which appear to be almost all males and Asians (Bouna-Pyrrou et al. 2018). This underlines a serious problem in the study of the GD as very few data relating to females, as well as the lack of demographic heterogeneity. Further, dimorphism is associated with psychiatric comorbidities (Tang and Koh 2017) and the development of poor mental health (Ciarrochi et al. 2016), which appears to be greater in female gamers. Probably, this is because there are important sex differences in the circuits that control attention, addictions, and psychiatric behaviors, both in their structure and in differentiated responses.

In these studies, the poor consideration of sex, the different ages of the players, the different types of games, and the mode of recruitment of subjects represent the limiting factors that could be operationally more easily overcome using an animal model, which would allow standardization of these parameters along with the combination of behavioral and molecular analyses performed in the different brain areas (Barrus and Winstanley 2017; Marraudino et al. 2022; Rafa et al. 2016).

Animal models that reflect some of the features described in GD patients could be a valuable tool for studying the behavioral and neurobiological characteristics of the disorder. However, at present, no GD-specific models have been developed so far. On the contrary, in the literature, there are several gambling models developed in rats that use electronic equipment, such as dispensers or screens (Barrus and Winstanley 2017; Rafa et al. 2016; Winstanley et al. 2011). Usually, there is an association of visual stimulus with a reward released by pushing a lever. Thus, there is no direct interaction with the screen, which represents only the visual part of the task. These rat models primarily focus on the animal’s ability to discriminate one more profitable choice over another while neglecting loss of control and the development of hyperactive behaviors. Moreover, most of them used male rats, considering the high incidence for gambling in men. Of these, only two studies correlate changes in neural activity following the development of this disorder. The first one revealed in two regions, prefrontal cortex (PrL) and the orbitofrontal cortex (OFC), an increase in the number of c-Fos positive cells (a neuronal activity marker); while, the second one, in the Paraventricular Nucleus of the Thalamus (PVT), showed a sex difference, with greater activation in males and a reduction in females (Koot et al. 2014).

The main purpose of these models is to understand the mechanisms underlying the loss of rational control over the game in the presence of random choices, that mimic slot machine use. Loss of control over gaming is also one of the main features of GD, as well as the hyperactivity and compulsivity under-studied in the rat models of gambling (Barrus and Winstanley 2017; Rafa et al. 2016; Winstanley et al. 2011). These two parameters are partially assessed by quantifying the frequency of lever pushing. Thus, there is neither an estimation of the duration of “play” nor an assessment of the type of play (hyperactive or non- hyperactive). Quantification of duration of play and type of play are very important behavioral parameters for assessing the loss control and hyperactive components in GD.

Another aspect that is little or completely absent in gambling models is the assessment of social context, another aspect that is highly compromised in GD patients. In recent years, social context or social interaction has been more studied, especially the positive effect it has on reducing self-administration or relapse in animal models of substance dependence (El Rawas et al. 2012; Fritz et al. 2011; Venniro et al. 2018). Thus, taking advantage of a new apparatus consisting of a touch-screen platform, the main purpose of this work is to propose a new rat model that closely reflect the behavioral phenotype observed in GD patients, allowing to evaluate the hyperactive component, loss of control and to investigate the possible positive effects of social context on the reduction of parameters associated with GD.

## Materials and methods

### Animals

Adult male (*n* = 4) and female (*n* = 8) Wistar Kyoto rats were purchased from Charles River (Charles River Laboratories Italia s.r.l., Milan, Italy). Rats were housed in standard conditions at 22 ± 2 °C, under a 12:12 light-dark cycle (lights on at 08:00 AM). Food (standard chow diet, VRF1, SDS -Charles River Laboratories) and water were provided *ad libitum* throughout the study. One male and two female rats (3-month-old) were housed together to achieve a successful mating. Obtained pups (37 males and 41 females) were sexed and weaned at postnatal day 28 (PND28) and were then housed into separated cages containing 4 same-sex rats.

### Experimental groups

After the selection phase (described below), selected rats (*n* = 30 males / 34 females) at PND40 were randomly divided into the following experimental groups:

- Control males (CON-M, *n* = 12);
- Control females (CON-F, *n* = 13);
- Male rats subjected to GD protocol (GD-M, *n* = 18);
- Female rats subjected to GD protocol (GD-F, *n* = 21).

The control groups underwent a 10 min/day period of adaptation to the apparatus, one week before the beginning of the testing phase. The GD group underwent the pre-training and training phase as described below.

Animal care and handling were according to the European Union Council Directive of 22nd September 2010 (2010/63/UE); all the procedures reported in the present study were approved by the Italian Ministry of Health (authorization n°1035/2020-PR). The experimental design conforms to the ARRIVE guidelines originally published by Kilkenny et al. in 2010 (Kilkenny et al., 2010).

### GD protocol

**Fig. 1:**
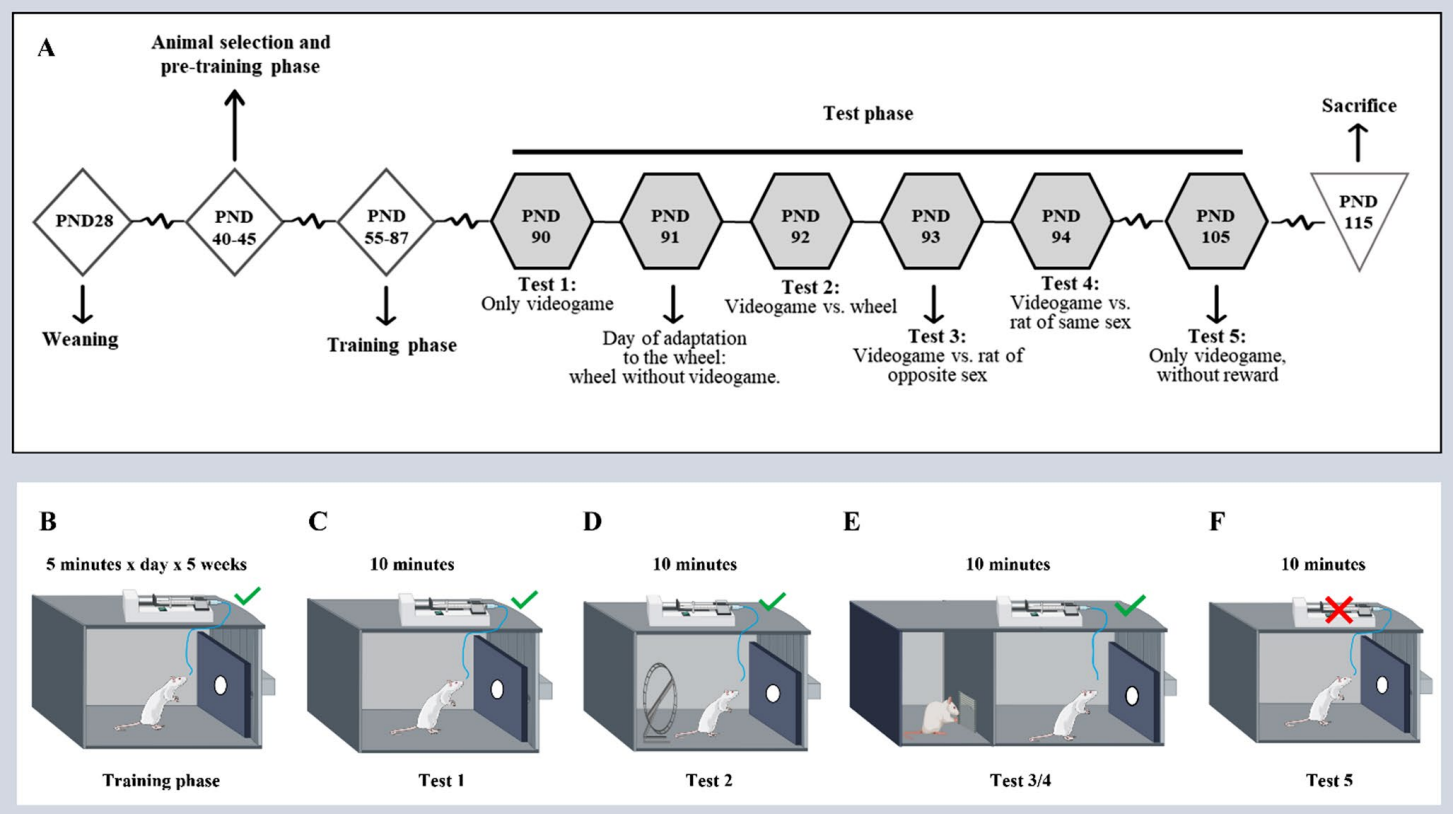
Experimental timeline. Schematic representations of (**(A)** experimental procedures’ timeline, **(B)** training and different stimuli during **(C-F)** Test 1 and Test 5 (videogame alone), **(D)** Test 2 (videogame vs. new object), and **(E)** Test 3 (videogame vs. sexual stimulus) and Test 4 (videogame vs. social stimulus). PND = Postnatal day

A schematic representation of the experimental timeline is reported in Fig. 1A.

### Behavioral apparatus

**Fig. 2:**
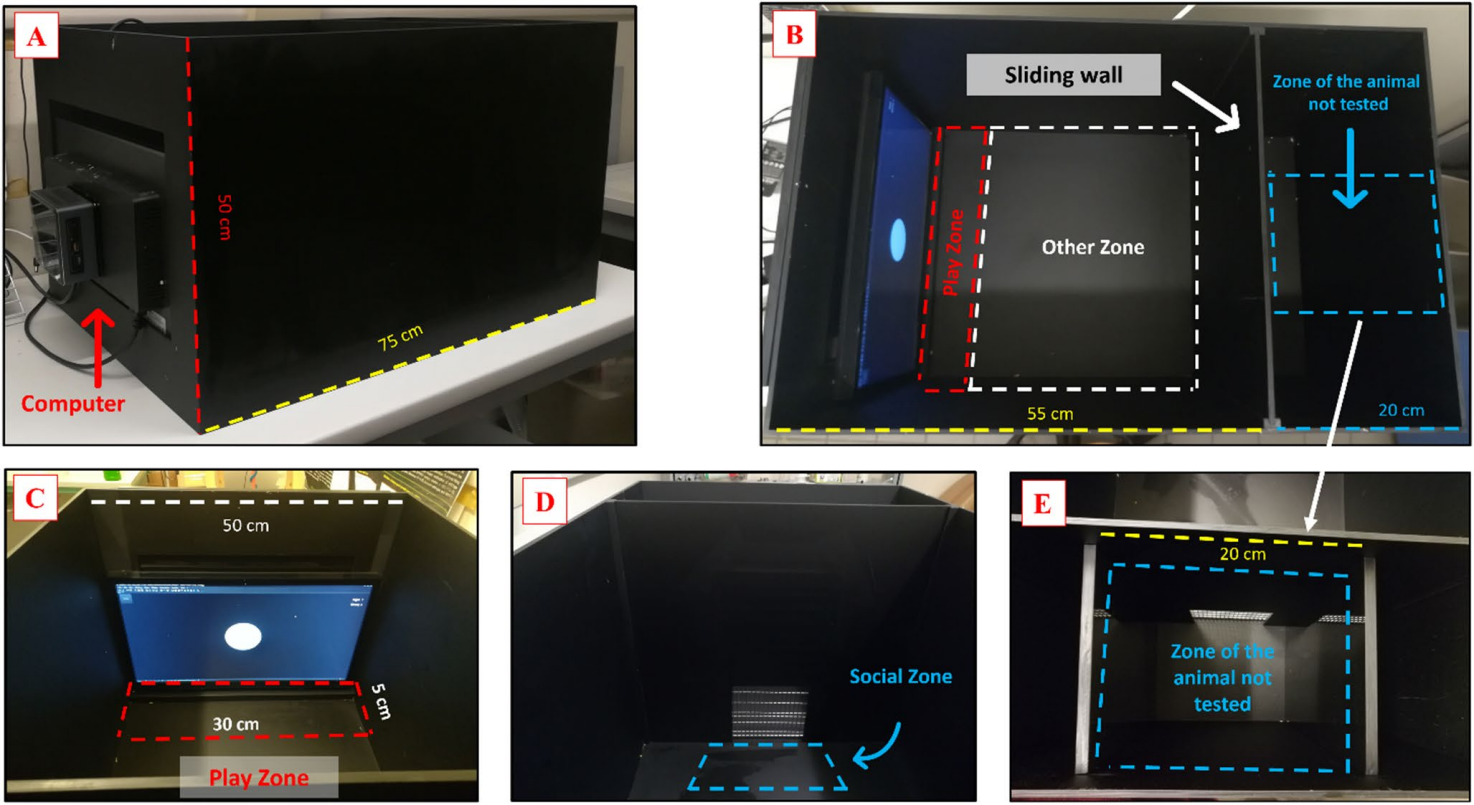
Behavioral apparatus. Photos of **(A)** the apparatus with **(B-E)** a graphic representation of the different zones (play zone, other zone, and social zone). The apparatus has **(B)** a movable panel that was replaced with **(D)** an open one during tests where social interaction is planned (Test 3–4). The red rectangle represents the play zone, the blue rectangle represents the social zone, and finally, the white rectangle represents the remaining zone of the arena

The GD protocol took place in a rectangular planned apparatus (50 × 55 × 50 cm) with a plastic base and plywood walls (Fig. 2). The apparatus was positioned 50 cm above the floor in a room illuminated with medium-low light using a house light, tempered to 22 ± 2 °C, and with a camera (Basler GenICam, acA 1300-60 gm) fixed on the roof to register (Media Recorder, Noldus, Wageningen, the Netherlands) the sessions.

In the center of the apparatus, there was a removable panel that allowed it to be divided into two areas: on the one hand, the play area (50 × 55 × 50 cm), on the other one (20 × 50 × 50 cm) an area in which different competitive stimuli were placed during the test 2, 3 and 4 (detailed below).

In the play area, a 50 × 30 cm touchscreen tablet (developed by UX experts from SCAI DOO.IT Group, Turin, Italy) was fixed on the shorter wall of the apparatus, with which the rats could interact freely. The area in front of the touchscreen tablet was defined as the play area (5 × 30 cm) (Fig. 2).

The game involved the fixed or random appearance of a white dot (4.5 cm in diameter) on the black screen. When the animal touched the dot correctly, it got a reward of 0.5 g of yogurt (Strawberry yogurt – no added sugar). Yogurt delivery followed a fixed ratio (FR) for the first 3 weeks of the training phase, and then it moved to a random ratio (RR) for the last 2 weeks of the training and for the Test phase.

### Selection of animals and pre-training phase

Starting from PND40, rats underwent the selection, pre-training, and training phases.

All animals participated in the selection phase. From PND40 to PND45, the spontaneous behavior of the animals was observed for 20 min in their home cages after 1 h of habituation in the behavior room. Exploratory parameters were used to select rats having showed high levels of anxiety toward a new environment (room in which they would perform the training period and the test period) or toward the operator. The assessment consisted of evaluating exploratory ability (movement inside the cage), fear of the operator, behaviors implemented to get the yogurt (leaning out of the cage) and freezing (Palanza 2001). The daily score value was assigned according to the percentage of time during which the rat performed that type of behavior (0 (< 20%); 1 (> 20/<40); 2 (> 40/<60); 3 (> 60/<80); 4 (> 80/<100). 17 was defined as the mean score obtained from the weekly average to all subjects. Animals with a five-day mean score of less than 17 were excluded from the study (M = 2, F = 3). All the other animals were randomly divided into the following groups: controls (M = 12, F = 13), which were not subjected to the next pre-training and training phases, and animals subjected to GD protocol (M = 18, F = 21).

In the pre-training phase, from PND48 to PND52, the animals underwent a period of adaptation to the apparatus of 5 min per day. The spontaneous behaviors of the animals were also observed in this phase. As before, the assessment consisted of evaluating exploratory ability, fear of the operator, behaviors implemented to get the yogurt, and freezing, assigning to each behavior a daily score (0 = never, 1 = little, 2 = enough; 3 = a lot; 4 = always). Animals that were frightened by the apparatus explored little or nothing and did not come close to the reward and displaying a five-day mean score of less than 17 were excluded from the experiment (M = 5, F = 4).

### Training phase

The training phase lasted 5 weeks (from PND55 to PND87), 5 days per week. The sessions took place always between 8:00 a.m. and 1:00 p.m. The rats were placed in the room with soft light (20 lx) 1 h before the beginning of the test.

In the first training week, during the first 3 days, the tablet was in OFF mode. On the last two days of the first week, the tablet was switched to ON mode with the videogame running, and the reward was dispensed with FR1 1:1 (1 correct touch = 1 reward). In the second week, the reward was dispensed with FR2 2:1, whereas for week 3 dispensing was with FR3 3:1. In the 4th and 5th week of training, the reward was dispensed in RR (random ratio).

Each day, the rats were evaluated for the different game-related parameters: time spent in front of the screen, interaction with the screen (both correct touch or not), following the dot, undertraining the connection between correct touch and reward, the number of touches made, and reward obtained.

A form was filled daily with reached scores in all evaluated parameters for each rat. In this form were indicated the different behaviors related to the game. Depending on the number of correct touches, a different score was assigned to each game-related behavior performed by the animal (time spent in front of the screen, interaction with the screen, following the dot, understanding the dot-reward link). The score assigned to each behavior (from the minimum of 1 to the maximum of 5) was related to the number of correct touches (0 = 0 correct touches; 1 = 1–5 correct touches; 2 = 6–10 correct touches; 3 = 11–15 correct touches; 4 = 16–20 correct touches; 5 ≥ 20 correct touches). The daily score was the sum of the scores obtained in each behavior plus the sum of the correct touches. In this way, it was also possible to evaluate the daily progression of the performance. Last, each week, a mean weekly score was assigned to each animal.

### Test

The test phase, lasting 6 consecutive days, from PND90 to PND95, aimed to understand if the animals have developed game addiction, testing their ability and interest in the videogame even in the absence of reward and in the presence of new stimuli (exploratory, sexual, or social). Thus, the tests were performed as follows (Fig. 1A):

#### - PND90: Test 1

The tablet was in ON mode with the videogame running (Fig. 1C*)*. The animal was allowed to choose whether to interact with the videogame to obtain the reward or to explore the apparatus. Tested animal was assessed for attachment (time spent in the play zone), duration (time spent interacting with the video game), and loss of control (number of correct touches, speed, and distance traveled in the play zone) shown during the play session.

#### - PND91: Day of adaptation to the new object (wheel)

The animal could explore the apparatus in the presence of the wheel, a hitherto unknown stimulus, with the tablet in OFF mode.

#### - PND92: Test 2

The condition was similar to the previous day (the day of adaptation to the wheel), but the tablet was switched ON with the videogame running. The animal was free to choose whether to play or to interact with the stimulus, and the same parameters of Test 1 were evaluated with the addition of all those behaviors expressed towards the wheel (exploration time and duration of the run on the wheel) (Fig. 1D).

#### - PND93: Test 3

The tablet was in ON mode with the videogame running. In addition, an unknown animal of the opposite sex (socio-sexual stimulus) was present in the apparatus (Fig. 1E). The animal was able to choose whether to interact with the video game or with the sexual stimulus. The time spent in the sexual zone (zone adjacent to the sexual stimulus), number, and total duration of sniffing, in addition to the same parameters evaluated in Test 1, were recorded.

#### PND94: Test 4

The test condition was similar to test 3, but a co-specific of the same sex (social stimulus) was present in the apparatus (Fig. 1E). The parameters assessed were the same as in Test 3.

#### PND95: Test 5

Differently from the previous tests, the animal was evaluated as in Test 1 but did not receive a reward, when it made the correct touches (Fig. 1F).

All females (both tester and no-tester ones) were tested during the estrus phase of the ovarian cycle in Test 3 or 4, when social interaction with a conspecific was planned. Thus, if the animal was not in the estrus phase, the test was postponed until the next day. The estrus phase was assessed by vaginal smear (Cora et al. 2015).

All tests lasted 10 min and were recorded using the camera attached to the roof at a distance of 2.5 m from the apparatus. The reward was dispensed in RR.

The following parameters were evaluated during the tests and were subsequently analyzed with EthoVision 8 (Noldus Information Technology; Noldus, Spink, & Tegelenbosch, 2001):

- Time (s) spent in the different zones of the arena (play zone and arena);
- Distance (cm) traveled (in different zones or total arena);
- Speed (cm/s) achieved (in different zones or total arena);
- Game-related behaviors: interaction with games (cumulative duration), correct touches (i.e., number of goals; frequency);
- Non-game-related behaviors: grooming (cumulative duration), protected rearing (frequency), and unprotected rearing (frequency);
- Wheel-related behaviors: time (s) spent exploring the wheel, time (s) spent running on the wheel;
- Behaviors related to socio-sexual or social interaction: time (s) spent sniffing.

### Fixation and tissue sampling

At PND115, a subset of rats, randomly selected (5 control males, 5 control females, 5 GD males, and 5 GD females), underwent a 10-minute play session were sacrificed 90 min later by deep irreversible anesthesia (intraperitoneal injection of Zoletil 100 mg/kg - Rompum 20 mg/kg) and transcardially perfused with 0.9% NaCl and then with 4% paraformaldehyde (PFA) solution (Tronel and Sara 2002). Females were sacrificed in the estrus phase, assessed by vaginal smears (Alboni et al. 2017; Cora et al. 2015; Di Micioni et al. 2017).

Brains were removed and stored in 4% PFA solution for 24 h, followed by several washes in 0.01 M phosphate-buffered saline (PBS). Finally, they were stored in 30% sucrose solution in 0.01 M PBS at + 4 °C, frozen in pre-cooled isopentane on dry ice at -35 °C and stored in a deep freezer at -80 °C until sectioning.

Brains (*n* = 5/group) were serially cut in the coronal plane at 30 μm thickness using a cryostat in three series. The sectioning plane was oriented to match the corresponding patterns to the coronal sections of the rat brain atlas (Paxinos and Watson 1998). Sections were collected in a cryoprotective solution and stored at -20 °C.

### c-Fos immunohistochemistry

The presence of c-Fos was detected by immunohistochemistry performed on free-floating sections from one series. Briefly, the sections were washed overnight in 0.01 M PBS at pH 7.3. The following day, sections were first incubated with a citrate buffer (citric acid 10 mM, 0.05% Tween, pH 6.0) previously heated at 95 °C for antigen retrieval and then washed three times in 0.01 M PBS. Next, the sections were washed in 0.01 M PBS containing 0.5% Triton X-100 for 30 min and then treated to inhibit endogenous peroxidase activity with a solution of 0.01 M PBS containing methanol/hydrogen peroxide for 20 min. Sections were incubated for 30 min with blocking solution containing normal goat serum (Vector Laboratories, Burlingame, CA, USA) and bovine serum albumin (Sigma-Aldrich, St. Louis, Missouri, USA) diluted in 0.01 M PBS containing 0.2% Triton, and then incubated two overnight at + 4 °C with polyclonal anti-c-Fos antibody (Cell Signaling Technology, Danvers, Massachusetts, USA; 9F6, Cat. #2250; Rabbit, 1:3.000) diluted in blocking solution. A biotinylated goat anti-rabbit secondary antibody (Vector Laboratories, Burlingame, CA, USA) diluted in 0.01 M PBS, pH 7.3–7.4, containing 0.2% Triton X-100 was then used at a dilution of 1:200 for 60 min at room temperature. The antigen-antibody reaction was revealed by 60 min incubation with avidin–peroxidase complex (Vectastain ABC Kit Elite, Vector Laboratories, Burlingame, CA, USA). The peroxidase activity was visualized with a solution containing 0.400 mg/ml 3,3-diamino-benzidine (Sigma-Aldrich, Milan, Italy) and 0.004% hydrogen peroxide in 0.05 M Tris–HCl buffer at pH 7.6. Sections were mounted on chromallum-coated slides, air-dried, cleared in xylene, and cover-slipped with New-Entellan mounting medium (Merck, Milano, Italy). This antibody was successfully used in previous studies (Cho et al. 2020; Netser et al. 2020; Zhou and Jia 2021). The specificity of this antiserum was previously assessed (Kovary and Bravo 1991), but, as a further control, we omitted the primary antiserum or the secondary biotinylated one and replaced it with 0.01 M PBS. Positive cell bodies were totally absent.

### c-Fos quantitative analysis

For quantitative analysis, selected standardized sections of brain areas summarized in Supplementary **Table 1**, were chosen according to the rat brain atlas (Paxinos and Watson 1998). Sections of comparable levels (see Supplementary **Table 1**) for each nucleus were acquired with Slide-Scanner Axioscan Z1 (ZEISS, Oberkochen, DE) both at low and high magnification (5x and 20x, respectively). Digital images were processed and analyzed by ImageJ (version 2.10/1.53c; Wayne Rasband, NIH, Bethesda, MD, USA). Measurements were performed within predetermined fields (region of interest, ROI), boxes of fixed size and shape that are inserted inside each labeled considered nucleus (see Supplementary **Table 1**). In particular, we counted the number of c-Fos-positive cells in all analyzed nuclei.

### Statistical analysis

Quantitative behavioral data was analyzed with SPSS 27 statistic software (SPSS Inc, Chicago, IL, USA) via two-way analysis of variance (ANOVA), with sex and condition (GD vs. CON) considered as independent variables. To understand whether the behavior of the GD groups was maintained across the different tests, we compared the behavioral parameters performed in Test 1 with the other tests, using three-way ANOVA (test, sex, and condition as independent variables) for repeated measures.

For the data from the last week of training, we used two-way ANOVA (day and sex as independent variables) for repeated measures to compare the behaviors of the GD groups on different days.

Either two-way ANOVA (as described for behaviors) or nested one-way (nested ANOVA) was used to quantify immunohistochemical data, when there were multiple data from the same brain area of the same animal. Using the nested ANOVA allowed us to account for within-sample variability (between observations of the same animal) and within- and between-group variability: group (CON vs. GD) was considered a fixed factor, while animals and observations (cells/sections) were considered random factors; sample sizes (N of animals) in the groups and N of observations per sample were kept the same. If the ANOVA was significant, the *post hoc* analysis was performed using Tuckey’s HSD (honestly significant difference) test.

Finally, to find out whether there was a significant correlation between time spent in the play zone and other behaviors (the speed and distance traveled in the play zone, duration of interaction with the tablet, and number of correct touches) during training and in Test 1, we used Person R Correlation.

The data are presented as mean ± SEM, and the differences between groups are considered significant for values of *p* < 0.05.

## Results

### Training

To determine whether animals undergoing the protocol-maintained game-related behaviors, we evaluated the last five days of training. The GD groups of both sexes, during the training phase, spent almost all the time in the play zone reducing the time spent in the remaining part of the apparatus (*p* < 0.001). Excluding some small daily variation, when comparing the different parings assessed, the rats of both sexes in the GD group kept both the distance traveled in the play zone, the time spent interacting with the game, and the number of correct touches constant.

Performing Person R Correlation, it was possible to find a significant correlation among different behavioral parameters. Increasing time spent in the play zone was positively correlated with increasing distance traveled (*p* < 0.001 for males; *p* = 0.041 for females) and velocity in the play zone (*p* < 0.001, for males; *p* = 0.005, for females), game interaction (*p* = 0.002 for males; *p* = 0.005 for females), and a number of correct touches (*p* < 0.001 for males; *p* = 0.013 for females).

### Test

Behavioral results obtained from the analysis of the different tests are summarized in Supplementary **Table 2.** The p-values present in the results come from Tuckey’s HSD.

#### Test 1

**Fig. 3:**
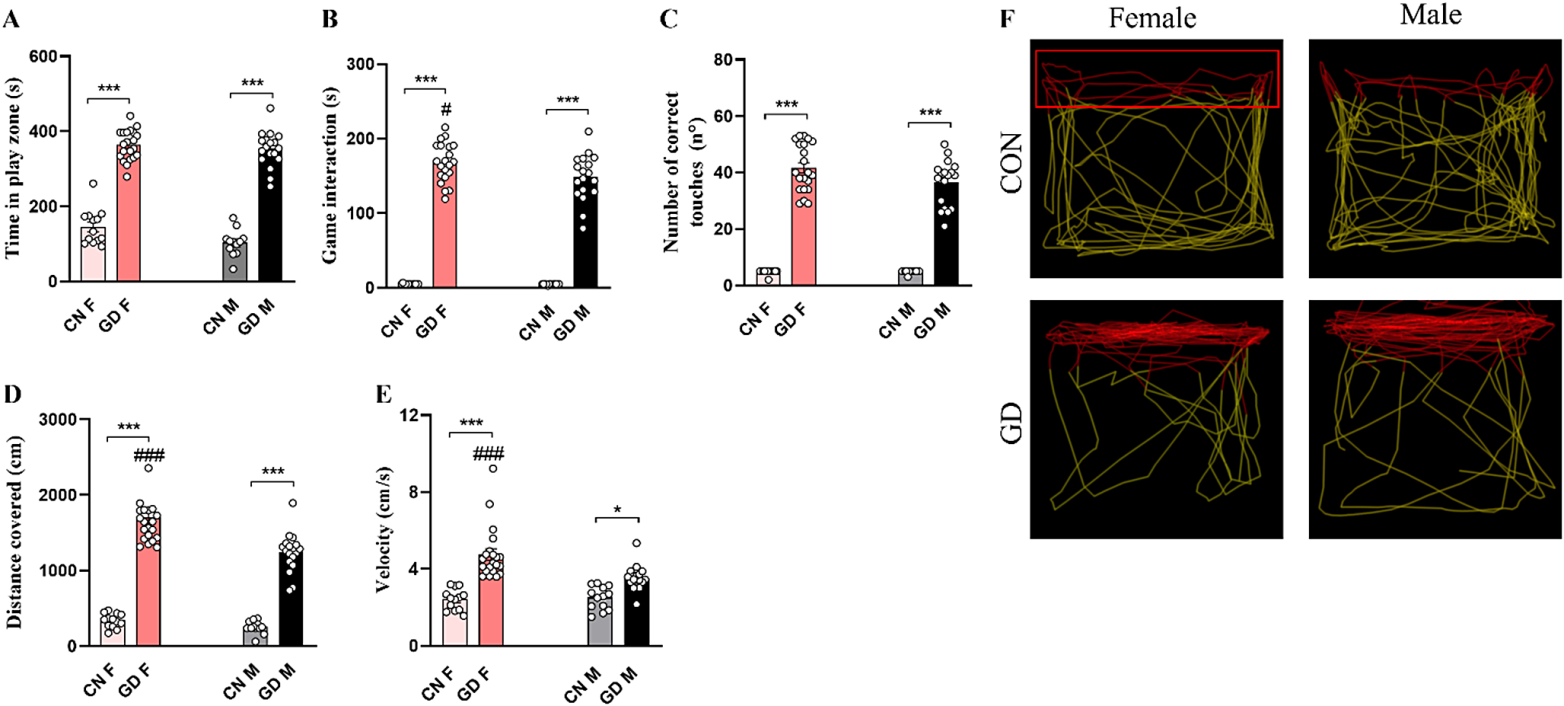
Analysis of the addictive behavior during Test 1 (in the presence of the reward). The upper histograms show **(A)** the time spent in the play zone, **(B)** the time spent in game interaction, and **(C)** the number of correct touches performed by the different groups. The lower histograms show **(D)** the distance traveled in the play zone, and (**E)** the mean velocity. (**F**) Representative images of the distance traveled by the different experimental groups in the total arena and within the play zone (red rectangle). Data are expressed as mean ± SEM. Two-way ANOVA followed by Tukey’s HSD test revealed a significant effect for *p* < 0.05 (* comparison between different groups; **#** comparison between sexes)

In Test 1, male and female GD animals spent significantly more time in the play zone, reducing that spent in the remaining part of the apparatus compared with same-sex control animals (Fig. 3A; CON-M vs. GD-M *p* < 0.001; CON-F vs. GD-F *p* < 0.001). Interestingly, while spending time in the play zone, GD groups of both sexes interacted more with the videogame compared to same-sex CON groups (Fig. 3B; CON-M vs. GD-M *p* < 0.001; CON-F vs. GD-F *p* < 0.001).

This increase in interaction with the videogame of GD was correlated with an increase in the number of correct touches (Fig. 3C; CON-M vs. GD-M *p* < 0.001; CON-F vs. GD-F *p* < 0.001), the distance covered (Fig. 3D CON-M vs. GD-M *p* < 0.001; CON-F vs. GD-F *p* < 0.001), and, only in females, also to a greater speed in the play zone (Fig. 3E; *p* < 0.0001) related to the same-sex CON group. Finally, in GD animals, males and females displayed a similar number of correct touches (Fig. 3C; *p* = 0.068). Conversely, GD animals displayed sexual differences in the game interaction (Fig. 3B; *p* = 0.036), the distance traveled (Fig. 3D; *p* < 0.0001), and the speed (Fig. 3E; *p* < 0.0001) in the play zone, with females displaying higher values compared to males.

In addition, the Person R Correlation showed a significant correlation both between time spent in the play zone and game interaction (*r* = 0.797, *p* < 0.0001 for males, *r* = 0.491, *p* = 0.024 for females) and with the number of touches (*r* = 0.815, *p* < 0.0001 for males, *r* = 0.505, *p* = 0.020 for females) in the GD groups.

#### Test 2

**Fig. 4:**
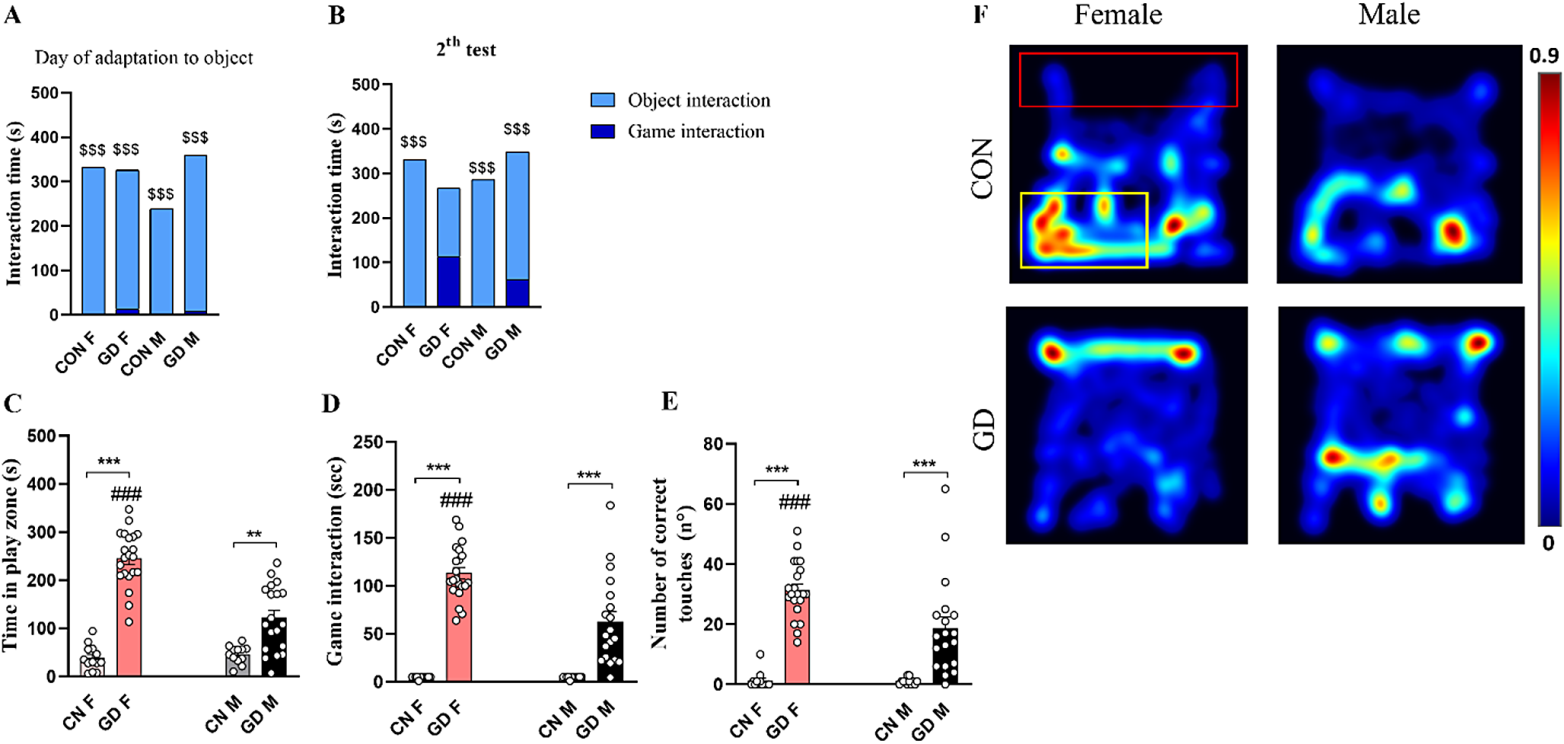
Analysis of behavioral data during the wheel habituation phase and Test 2. Graph in **(A)** shows the comparison between the time spent interacting with the wheel (evaluated as time spent sniffing the wheel) and the time spent interacting with the tablet, in OFF mode, on the wheel habituation day of the different groups, while graph in **(B)** depicts that obtained on Test 2 day when the tablet was in ON mode and the videogame was running. The histograms in the lower panel show **(C)** the time spent in the play zone, **(D)** the time spent in game interaction, and **(E)** the number of correct touches performed by CON and GD animals during Test 2. **(F)** Representative Heatmaps of the time spent by the different experimental groups in the different zones of the arena (play zone delimited by the red rectangle and area of the wheel delimited by the yellow one). Data are expressed as mean ± SEM. Two-way ANOVA followed by Tukey’s HSD test revealed a significant effect of protocol for *p* < 0.05 (*comparison between different groups; **#** comparison between sexes). Person R Correlation significant for *p* < 0.05 (**$** comparison between Test 1 and Test 4). CON = control; GD = gaming disorder

On the second day, the wheel fit took place. During the adaptation to the wheel, the test always lasted 10 min, the tablet was in OFF mode, and the animal could explore and interact with the wheel. CON animals showed a sex difference in wheel exploration (evaluated as time spent sniffing): females explored more than males (Fig. 4A; *p* < 0.0001), and this sex difference was not maintained in the GD groups (Fig. 4A; *p* = 0.071). Comparing same-sex animals from the different experimental groups, significant differences was found only in males. In fact, CON-M spent less time on the exploration of the wheel than GD-M (Fig. 4A; *p* < 0.0001). Despite the videogame being in OFF mode, the GD-F group interacted more with the screen than the CON-F group (Fig. 4A; *p* = 0.272). However, no differences were found among the GD groups (Fig. 4A; *p* = 0.626). In addition, all experimental groups spend more time interacting with the wheel than the tablet (Fig. 4A; *p* < 0.0001).

During Test 2, when the tablet was switched to the ON mode and the videogame was running, the GD groups increased the time spent interacting with it compared to same-sex CON groups (Fig. 4B; CON-M vs. GD-M, *p* < 0.0001; CON-F vs. GD-F, *p* < 0.0001). Excluding the GD-F group (*p* = 0.075), all groups preferred to interact with the wheel to the game (Fig. 4B; *p* < 0.0001).

In Test 2, compared to the previous day of wheel habit, the video game was in the ON mode. This had a different effect on the assessments analyzed. The GD animals spent more time in the play zone than the same-sex CON groups (Fig. 4C; CON-M vs. GD-M, *p* = 0.001; CON-F vs. GD-F, *p* < 0.0001). Comparing the two sexes, CON groups showed no difference in the time spent in the play zone (Fig. 4C; *p* = 0.991), while GD-F spent more time in this zone than GD-M (Fig. 4C; *p* < 0.0001). The video game in ON mode also increased the interaction time with the screen compared with the day of the adaptation when the tablet was switched OFF in both GD groups compared to same-sex CON (Fig. 4D; *p* < 0.0001). Moreover, the GD-F showed more interaction with the game both in terms of time spent playing (Fig. 4D; *p* < 0.001) and number of correct touches than the GD-M (Fig. 4E; *p* < 0.001). This sex difference was not present in the CON groups (Fig. 4D-E; *p* = 1.000).

Comparing Test 1 and Test 2, the presence of the new object (i.e., the wheel) influenced all parameters assessed in the GD groups. GD groups of both sexes showed a significant reduction in the time spent in the play zone (CON-M vs. GD-M, *p* < 0.001; CON-F vs. GD-F, *p* < 0.001) along with a reduction in both the time spent interacting with the tablet (CON-M vs. GD-M, *p* < 0.001; CON-F vs. GD-F, *p* < 0.001) and the number of correct touches (CON-M vs. GD-M, *p* < 0.001; CON-F vs. GD-F, *p* < 0.008).

#### Test 3

**Fig. 5:**
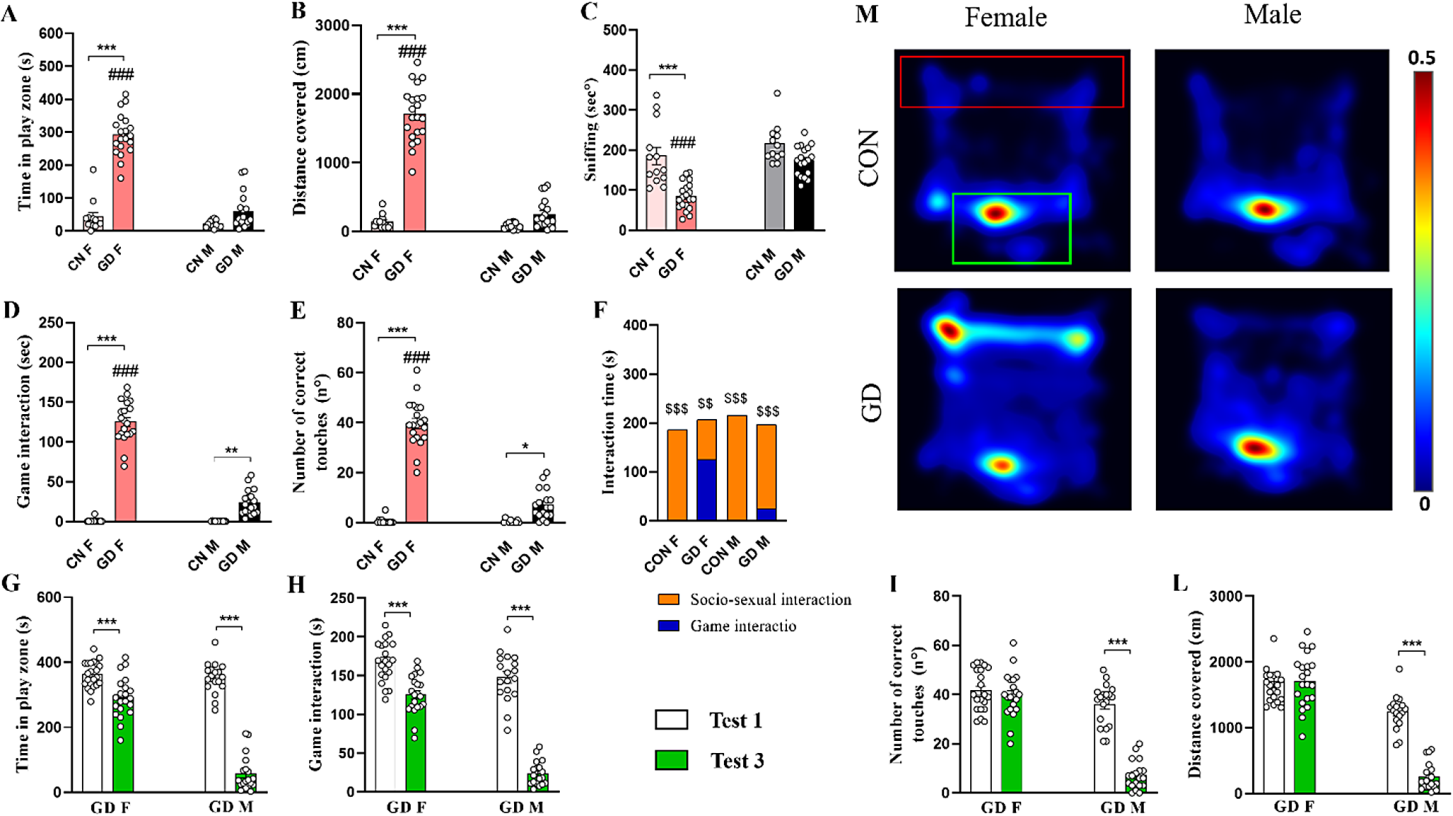
Analysis of behavioral data during Test 3. Histograms show **(A)** time spent in the play zone, **(B)** distance traveled in the play zone, **(C)** time spent in sniffing behavior, **(D)** time spent in game interaction, **(E)** the number of correct touches during play, and **(F)** the time (expressed as cumulative duration) spent in sniffing behavior on tester animal vs. play time during Test 3 (i.e., in the presence of an animal of the opposite sex). **(G)** Representative Heatmaps of the time spent by the different experimental groups in the different zones of the arena (play zone delimited by the red rectangle and social zone delimited by the green one). Comparison of **(G**) time spent in the play zone, **(H)** time spent in game interaction, **(I)** number of correct touches, and **(L)** distance traveled between Test 1 and Test 3. **(M)** Representative Heatmaps of the time spent by the different experimental groups in the different zones of the arena (play zone delimited by the red rectangle and social zone delimited by the green one). Data are expressed as mean ± SEM. Two-way ANOVA followed by Tukey’s HSD test revealed a significant effect of protocol for *p* < 0.05 (*comparison between different groups; **#** comparison between sexes). Person R Correlation significant for *p* < 0.05(**$** comparison between Test 1 and Test 3). CON = control; GD = gaming disorder

The presence of a sexual stimulus (opposite-sex no-tester rat) in the apparatus influenced differentially males and females during the test session. In many of the parameters evaluated, GD-M in the presence of a female behaved similarly to CON-M. In fact, time spent in the play zone (Fig. 5A; *p* = 0.195), distance traveled in the play zone (Fig. 5B; p = 0.321), and socio-sexual interaction (Fig. 5C; *p* = 0.107) did not differ compared to the CON-M. Instead, in females, the GD protocol produced a different effect among the different experimental groups.

GD-F showed a significant reduction in comparison to CON-F in socio-sexual interaction (Fig. 5C; *p* < 0.0001) but a significant increase in time spent in game interaction (Fig. 5D; *p* < 0.0001) and time spent in the play zone (Fig. 5A *p* < 0.0001). Furthermore, sex also influenced the performance in the duration of the game as well as for the correct number of touches in GD groups, but not in CON animals. Comparing males and females in the same group, except for the CON group (*p* = 0.738), GD-F spent more time in the play zone (Fig. 5A, *p* < 0.0001) and game interaction (Fig. 5D, *p* < 0.0001) than males, accumulating even a greater number of correct touches (Fig. 5E, *p* < 0.0001).

Furthermore, when comparing time spent game interaction to that spent interacting with a conspecific of the opposite sex, CONs and GD-M groups preferred socio-sexual interaction (Fig. 5F, *p* < 0.0001), while GD-F preferred playing (Fig. 5F, *p* = 0.002).

Comparing the time spent in gaming interaction obtained in this test to that observed in Test 1, revealed that the presence of the socio-sexual stimulus reduced play-related behaviors regardless of sex in GD groups. In fact, compared to Test 1, the GD groups reduced both times spent in the play zone (Fig. 5G; *p* < 0.0001, for males; *p* < 0.0001, for females) and game interaction (Fig. 5H; *p* < 0.0001, for males; *p* < 0.0001, for females). Interestingly, GD-M group significantly reduced the number of correct touches performed in Test 3 compared to Test 1 (Fig. 5I; *p* < 0.0001), while the GD-F group did not show any same difference (Fig. 5; *p* = 0.995). The GD-F group did not show any difference in the distance traveled in the arena between Test 1 and 3 (Fig. 5L; *p* = 1.000). However, they displayed an increase of the mean velocity in Test 3 (*p* = 0.016). In contrast, the GD-M group significantly reduced both parameters in Test 3 compared to test 1 (Fig. 5L; *p* < 0.0001, for distance; *p* < 0.0001, for velocity).

#### Test 4

**Fig. 6:**
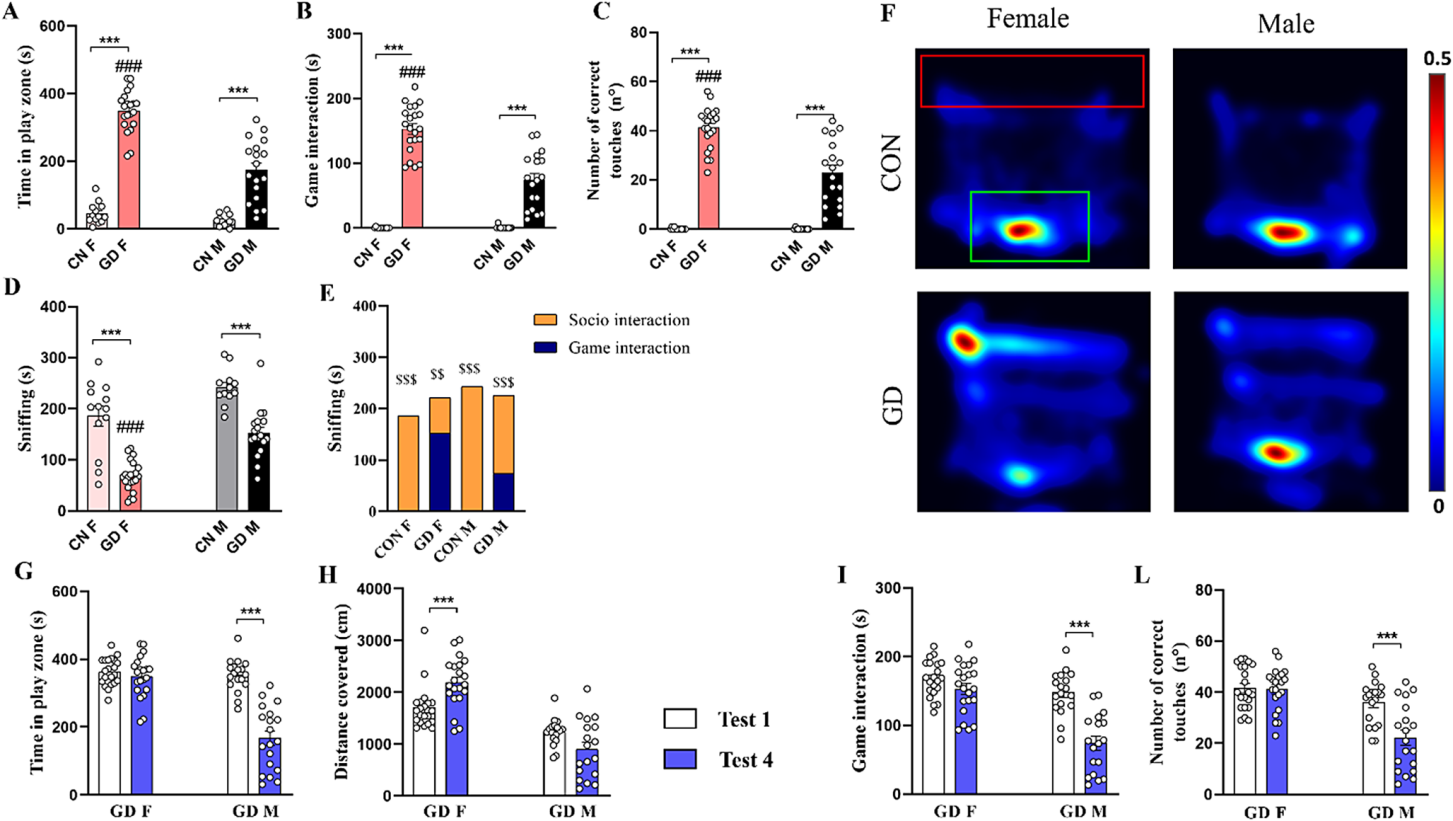
Analysis of behavioral data during Test 4. Histograms show **(A)** time spent in the play zone, **(B)** time spent in game interaction, **(C)** the number of correct touches during play, and **(D**) the time spent sniffing behavior towards no-tester rat. **(E)** Comparison between the time (expressed as cumulative duration) spent in sniffing behavior towards no-tester animal vs. time spent in game interaction during Test 4 (i.e., in the presence of an animal of the same sex). **(F)** Representative Heatmaps of the time spent by the different experimental groups in the different zones of the arena (play zone delimited by the red rectangle and social zone delimited by the green one). Comparison of **(G)** time spent in the play zone, **(H)** distance traveled, **(I)** time spent in game interaction, and **(L)** number of correct touches between Test 1 and Test 4. Data are expressed as mean ± SEM. Tukey’s test revealed a significant effect of protocol for *p* < 0.05 (*comparison between different groups; **#** comparison between sexes). Person R Correlation significant for *p* < 0.05(**$** comparison between Test 1 and Test 4). CON = control; GD = gaming disorder

In Test 4, the presence of a social stimulus (same-sex no-tester rat) had a less strong effect with respect to sexual stimulus on gaming. During this test, as before, GD-rats spent more time in the play zone compared to same-sex controls (Fig. 6A; CON-M vs. GD-M, *p* < 0.0001; CON-F vs. GD-F, *p* < 0.0001). In particular, GD-F spent more time in the play zone (Fig. 6A; *p* < 0.0001), game interaction (Fig. 6B; *p* < 0.0001**)** and performed better in terms of correct touches (Fig. 6C; *p* < 0.0001**)** than GD-M. As found previously in Test 3, also in this test, the new stimulus mainly interferes with gaming behavior. In fact, GD rats spent less time in sniffing behavior compared to same-sex controls (Fig. 6D; CON-M vs. GD-M, *p* < 0.0001; CON-F vs. GD-F, *p* < 0.0001). Interestingly, GD-F spent significantly less time in sniffing behavior compared to GD-M (Fig. 6D; *p* < 0.0001). Indeed, when comparing time spent playing to that spent in social interaction *(*Fig. 6E), the CONs (*p* < 0.0001, for both males and females) and the GD-M (*p* < 0.0001) groups preferred interaction with same-sex conspecifics. On the contrary, GD-F spent more time game interaction (Fig. 6E; *p* < 0.0001).

Differently to Test 3, in Test 4 the social stimulus produced a different sex-dependent effect. Comparing Test 4 with Test 1, the presence of a rat of the same sex reduced game-related behavioral parameters only in the GD-M group. In fact, GD-M displayed a significant decrease in the time spent in the play zone (Fig. 6G; *p* < 0.0001), time spent playing (Fig. 6I; *p* < 0.0001), and the number of touches (Fig. 6L; *p* < 0.0001) in Test 4 compared to Test 1. In contrast, the GD-F group shows no difference between the two tests (Fig. 6G-I-L; *p* = 0.987, for the time spent in the play zone; *p* = 0.622, for the time spent playing; *p* = 1.000, for the number of correct touches).

#### Test 5

**Fig. 7:**
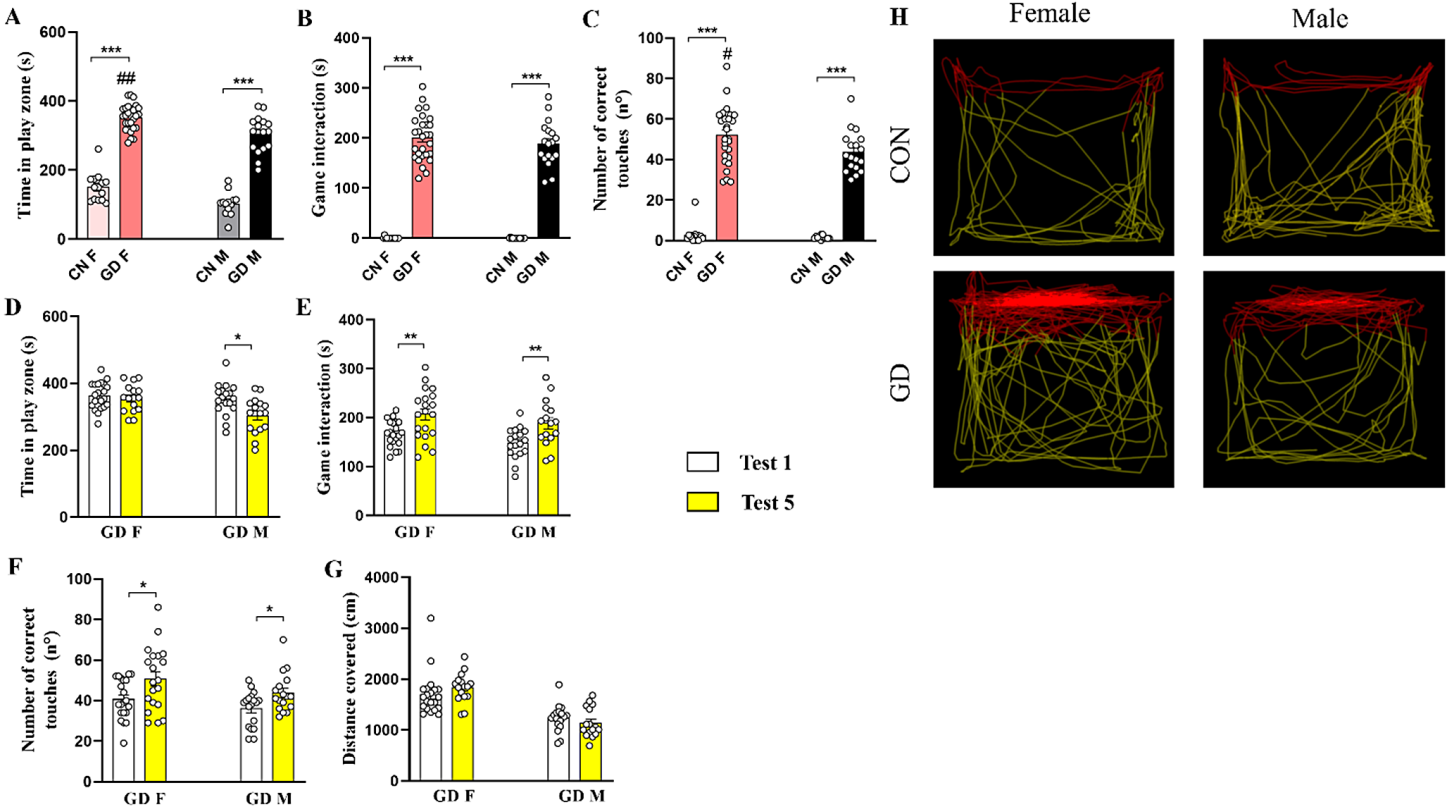
Analysis of the addictive behavior during Test 5 (in absence of the reward). The upper histograms show**(A)** the time spent in play zone, **(B)** the time in game interaction and **(C)** the number of correct touches performed during play. The lower histograms show the comparison between Test 1 (i.e., in presence of the reward) and Test 5 (i.e., in absence of the reward) in terms of **(D)** the time spent in play zone, **(E)** time spent in game interaction, **(F)** the number of correct touches and **(G)** distance travelled in the play zone. (**H**) Representative images of the distance travelled by the different experimental groups in the different zones of the arena (red path for distance travelled in the play zone and yellow path for that travelled in the remaining part of the arena). Data are expressed as mean ± SEM. Two-way ANOVA followed by Tukey’s HSD test revealed a significant effect of protocol for *p* < 0.05 (* comparison between different groups; **#** comparison between sexes). Person R Correlation significant for *p* < 0.05($ comparison between Test 1 and Test 5). CON = control; GD = gaming disorder

In this last test, we analyzed interaction with the videogame in the absence of the reward. During Test 5, as before in Test 1, GD rats spent more time in the play zone (Fig. 7A; CON-M vs. GD-M, *p* < 0.0001; CON-F vs. GD-F, *p* < 0.0001) and game interaction (Fig. 7B; CON-M vs. GD-M, *p* < 0.0001; CON-F vs. GD-F, *p* < 0.0001) than the CON groups. Moreover, they also increased the number of correct touches compared to same-sex CONs (Fig. 7C; CON-M vs. GD-M, *p* < 0.0001; CON-F vs. GD-F, *p* < 0.0001**)**. In addition, the GD-F group showed a difference in the time spent in the play zone (Fig. 7A; *p* = 0.002) and in the number of correct touches (Fig. 7C; *p* < 0.0001), but not in the time spent game interaction (Fig. 7B; *p* = 0.689) compared with GD-M. Instead, comparing Test 1 with Test 5, it is interesting to note that the absence of reward induced a significant increase in game interaction only in GD groups ***(***Fig. 7E; for males, *p* = 0.020, for females, *p* = 0.013) and only GD-F, in the number of correct touches (Fig. 7F, *p* = 0.027).

### c-Fos immunoreactivity

The analysis of the immunoreactivity (ir) for c-Fos is summarized in Supplementary ***Table 3***. Here we show some relevant results.

#### Inter-individual decision-making circuits

**Fig. 8:**
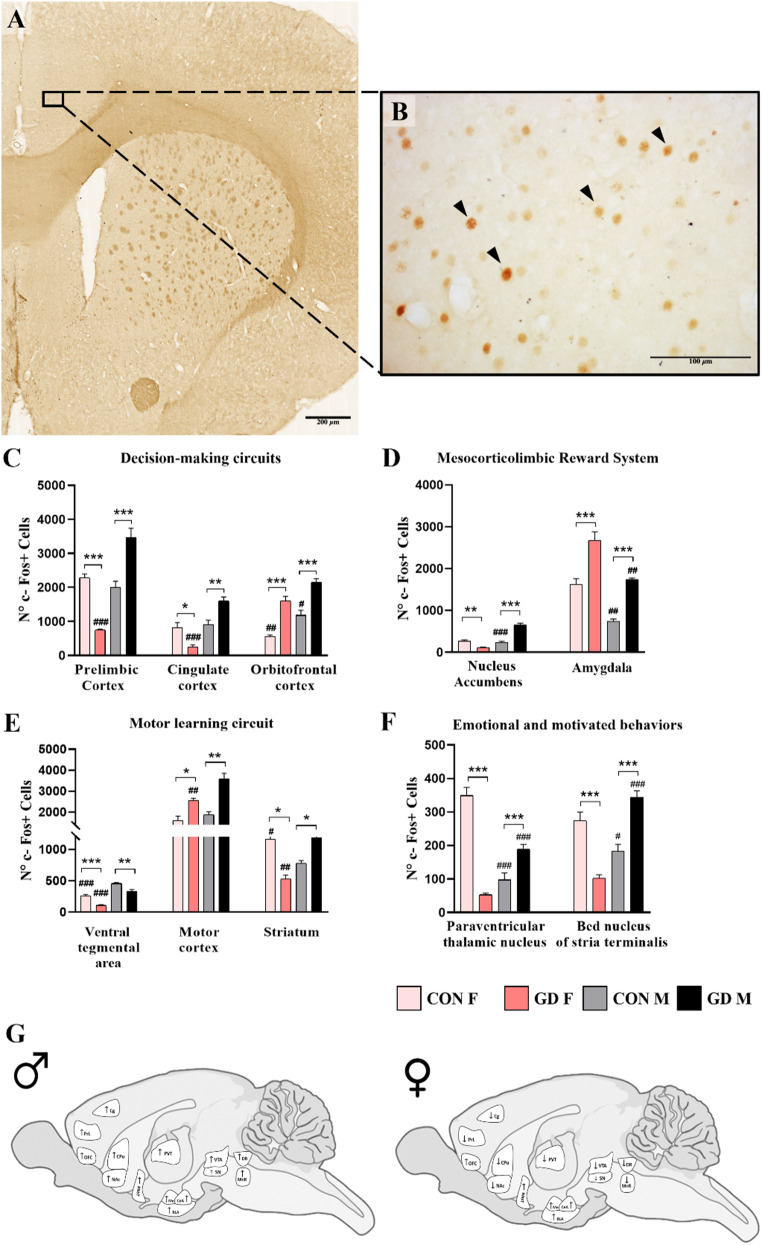
Analysis of c-Fos immunoreactivity in the brain circuits related to addictive behavior in the control and GD animals. Representative images of the c-Fos immunoreactivity in a coronal brain section from a control male rat at **(A)** 20x magnification (scale bar = 200 μm) and **(B)** 40x magnification (scale bar = 100 μm). Quantification of the number of c-Fos-positive cells in **(C)** decision-making circuit, **(D)** mesocorticolimbic reward system, **(E)** motor learning circuit, and **(F)** emotional and motivational circuit. **(G)** Images show the different areas involved in GD, that display higher (↑) or lower (↓) c-Fos-ir in the GD animals compared to control animals of the same sex. Data are expressed as mean ± SEM. Two-way ANOVA followed by Tukey’s HSD test was used to compare the different number of c-Fos positive cells in PrL, Cg, OFC, NAc. For the remaining areas (VTA, PVT, BNST, Striatum, and Amygdala), where more than one section per animal was present, nested ANOVA followed by Tukey’s HSD test was used. a significant effect for *p* < 0.05 (* comparison between different groups; # comparison between sexes)

The decision-making process is highly complex and inter-individually variable (Rivalan et al. 2011). It needs the integration of a wide set of executive functions, which involve different brain regions (Rivalan et al. 2011). Particularly relevant for this process are the orbitofrontal cortex (OFC), the prelimbic (PrL), and the cingulated (Cg) cortex (Rivalan et al. 2011). Within the control groups, only in the OFC, the c-Fos-ir was sexually dimorphic, with higher expression in males compared to females (Fig. 8C; *p* = 0.007). No sexual differences were found in the PrL (*p* = 0.654) or in the Cg (*p* = 0.943) (Fig. 8C). The dimorphism in OFC was maintained in GD groups (*p* = 0.018). However, the c-Fos-ir in GD rats increased in comparison to same-sex CON groups *(*Fig. 8C; CON-M vs. GD-M, *p* < 0.001; CON-F vs. GD-F, *p* < 0.001). Also, within the PrL, c-Fos-ir was differently affected in GD males and females compared to the controls, showing an increase in the males (*p* < 0.001) and a decrease in the females (*p* < 0.001). Last, in the Cg, GD-F displayed a decreased ir compared to CON-F (*p* = 0.004), whereas GD-M showed an increased ir compared to CON-M (*p* = 0.001). This led to a sexually dimorphic expression of c-Fos among GD-animals, with males showing greater ir compared to females (*p* < 0.0001).

### Mesocorticolimbic reward system

An important circuit for GD is the mesocorticolimbic reward system, which includes the nucleus accumbens (Acb) and the OFC (Chen et al. 2016). The c-Fos analysis showed that, while in the CON groups, the immunoreactivity in NAc did not show sexual dimorphism (Fig. 8D; *p* = 0.843), in GD animals, males presented greater c-Fos-ir than females (Fig. 8D; *p* < 0.0001). Moreover, GD-M showed an increased c-Fos-ir (*p* < 0.0001) compared to CON-M, while GD-F displayed a decreased c-Fos-ir in comparison to CON-F (*p* = 0.008) (Fig. 8D). The c-Fos expression in OFC was sexually dimorphic in control animals, with higher ir in males compared to females (*p* = 0.007). This dimorphism was maintained in GD groups (*p* = 0.018). However, the c-Fos immunoreactivity in GD animals increased in comparison to same-sex CON groups (Fig. 8C; CON-M vs. GD-M, *p* < 0.001; CON-F vs. GD-F, *p* < 0.001).

#### Motor learning circuit

Motor learning is considered the ability to adapt movement to changing environmental stimuli (Peters et al. 2017). The central component of motor-related circuits is the motor cortex, while particularly important in learning are its connections with the striatum and its modulation by ventral tegmental area (VTA) neurons (Peters et al. 2017). GD animals learned how to play with the tablet, acquiring the movement of touch to achieve goals. Interestingly, we found that in the primary motor cortex (M1) GD animals, unlike controls, displayed a sexual dimorphism, with males showing greater activation compared to females (*p* = 0.009). Furthermore, both GD groups displayed higher c-Fos-ir compared to same-sex control (Fig. 8E; CON-M vs. GD-M *p* < 0.001; CON-F vs. GD-F *p* = 0.015).

In the VTA, c-Fos-ir is sexually dimorphic in CON animals, with males showing higher ir compared to females (*p* = 0.005). This difference is maintained among the GD groups (*p* = 0.002). However, GD caused a reduction in c-Fos-ir in both GD-M (*p* = 0.032) and GD-F (*p* = 0.014) compared to same-sex controls (Fig. 8E). A similar effect is present in the substantia nigra (SN). Both groups show a sex difference (for CON, *p* < 0.001; for GD, *p* < 0.001) with a higher number of cells in males than in females. As in VTA, the GD group shows a higher number of c-Fos positive cells than the same-sex control group (for males, *p* = 0.001; for females, *p* < 0.001). Lastly, in the Dorsal Striatum, we observed higher c-Fos-ir in CON-F compared to CON-M (*p* = 0.003), but this sexual dimorphism was completely reversed in the GD groups (*p* < 0.001). In fact, GD-M displayed an increased ir compared to CON-M (*p* = 0.001), while GD-F showed a decreased ir compared to CON-F (*p* < 0.001) (Fig. 8E). While dividing the striatum into lateral and medial striatum, sex difference remains the same among both control (for lateral striatum, *p* = 0.013; for medial striatum, *p* = 0.003) and GD groups (for lateral striatum, *p* < 0.001; for dorsal striatum, *p* = 0.001). Also, in this subdivision of the striatum, the GD-F group shows a significant reduction compared with the CON-F group in both the lateral (*p* < 0.001) and dorsal striatum (*p* < 0.001). In contrast, the GD-M group, compared with the CON-M group, shows a significant increase in the expression of the number of c-Fos-positive cells in the lateral striatum (*p* < 0.001) and in the dorsal striatum (*p* = 0.036).

#### Regions related to anxiety, stress, and emotional and motivated behaviors

Response to stress stimuli and emotional and motivated behaviors are highly controlled by the paraventricular thalamic nucleus (PVT) and its connections with the Bed Nucleus of Stria Terminalis (BNST) and amygdala (Kirouac 2021). Interestingly in these regions, we found a sexually dimorphic expression of c-Fos, higher in females compared to males, which was completely reversed by GD (Supplementary ***Table 3***). Specifically, in the PVT, we observed higher c-Fos-ir in CON-F compared to CON-M (*p* < 0.0001), but the sexual dimorphism was reversed in the GD groups (*p* = 0.0005). In fact, GD-M displayed an increased ir compared to CON-M (*p* = 0.008), while GD-F showed a decreased ir compared to CON-F (*p* < 0.0001) (Fig. 8F). Within the BNST, CON-F showed higher c-Fos-ir compared to CON-M (*p* = 0.007), but the sexual dimorphism was reversed in GD groups, where GD-M displayed higher ir compared to GD-F (*p* < 0.0001). In fact, GD-M showed increased expression compared to the same-sex CON group (*p* < 0.001), whereas in GD-F c-Fos-ir was decreased compared to CON-F (*p* = 0.001) (Fig. 8F). In the amygdala, CON-M showed higher expression compared to CON-F (*p* = 0.001), while among the GD animals there is no difference (*p* = 0.285) (Fig. 8D). In particular, in GD animals, c-Fos-ir was increased compared to CON (Fig. 8D; GD-F vs. CON-F *p* < 0.001; GD-M vs. CON-M *p* = 0.035).

## Discussion

GD is a recognized mental health condition that urgently needs new therapeutic approaches. However, the research is limited and presents some bias. The possibility of being able to take advantage of an animal model could help the understanding of the core behavioral and neurobiological features present in GD, including loss of control and sex differences.

The new model here proposed resembles some of the specific behavioral characteristics observed in GD patients, such as loss of control over play accompanied by compulsive and hyperactive behavior (Lopez-Fernandez et al. 2019; Marraudino et al. 2022).

After 5 weeks of training, the rats subjected to GD protocol develop those patient-like traits, and the behavioral phenotype is maintained consistently over time. In fact, it does not decrease after stop periods but it does in the absence of reward (yogurt delivery). The loss control and hyperactivity components, measured by the number of correct touches, speed, and distance traveled in the play zone, are more strongly observed when the animals are in the play zone (near the tablet). Moreover, they are positively associated with the time spent in the play zone and the behaviors performed during the task. These aspects are completely absent in the control groups. During the task, this pattern does not show a sex difference except when rats are exposed to social context. In fact, in the presence of a social stimulus (same-sex no-tester rat), males show a reduction in behaviors associated with play, while females continue to prefer play to social interaction, possibly due to the alteration of reward circuits which are closely related to both social behavior and mental disorders.

At present, GD lacks an experimental model, and our schedule to induce GD in rats shows several advantages compared to other addiction-related models:

- no stressogenic deprivation (i.e., no caloric restriction or social isolation is used), differently from others (Barrus and Winstanley 2017; Di Ciano and Le Foll 2016; Ishii et al. 2018; Rafa et al. 2016; Rygula et al. 2012; Winstanley et al. 2011);
- developing a propensity to use electronic devices, this is similar to other gambling models (Barrus and Winstanley 2017; Ishii et al. 2012; Rafa et al. 2016; Rygula et al. 2012; van den Bos et al. 2006), but unlike others, the used task prompts the rats to interact as much as possible with the screen, because obtaining the reward is directly related to the correct touch of the moving stimulus;
- the use of a much larger apparatus (50 × 55 × 50 cm) than those used in gambling models (30.5 × 25.9 × 30.5 cm) (Barrus and Winstanley 2017) allows the development and quantification of the motor component in relation to the task, which is not entirely counted in other studies;
- the use of both sexes. Excluding the work of Ishii et al. (Ishii et al. 2018), this aspect is almost absent in most of the research done on gambling using rat models. The choice to exclude the female sex is justified by the higher incidence of addiction in men (Gartner et al. 2022). In recent years, numerous works showed that the incidence of the development of these mental disorders is similar between the sexes and that females manifest stronger symptoms than men (Lucas et al. 2023; Quigley et al. 2021). Enhancing the construct validity of our GD model, we observed that female rats, like male ones, show the same behavioral phenotype that remains stable even in different settings;
- the evaluation of the social component, another unexplored feature in other gambling models.

The addition of this last component strongly characterizes and differentiates our model from others. The GD group presents a reduction in social interaction in Test 3 (competitive socio-sexual stimulus) and 4 (competitive social stimulus) compared to controls, thus maintaining an inclination towards playing a videogame. However, GD rats display a sex-dependent reduction in addiction-related behaviors, demonstrating that, as happens in rat models of substance abuse, also in our protocol hyperactivity and seeking behaviors are reduced in the presence of a social reward (Test 3 and 4) (El Rawas et al. 2012; Fritz et al. 2011; Venniro et al. 2018).

Also, it is important to underline that, in addition to the establishment of harmful behaviors, GD patients present changes in brain activity within different circuits involved in the control of different aspects of addiction. For this reason, in our work, besides the behavioral evaluations, the brain areas activated during play were investigated, focusing on nuclei belonging to GD-relevant circuits, i.e., decision-making (Rivalan et al. 2011), mesocorticolimbic reward system (J.-T. Zhang et al. 2016), motor learning (Peters et al. 2017), and regions related to anxiety, stress, and emotional and motivated behaviors (Kirouac 2021). Our results support the idea that the development of GD led to changes in the activation of all the circuits mentioned above. Moreover, the sexual dimorphism in c-Fos-ir described could reflect some of the behavioral differences previously discussed.

There are only two works evaluating the expression of c-Fos in gambling (Ishii et al. 2018) or gambling-related (Koot et al. 2014) rat model. The first considers only the PrL and OFC of male rats, whose c-Fos expression is increased (Ishii et al. 2018), as we observed in our GD-male rats. The second assesses c-Fos expression only in the PV in both sexes (Koot et al. 2014), showing a sex difference in line with our findings.

Comparing this model with other preclinical models of Gambling or addiction, it revealed similar behavioral traits, such as the development of a hyperactive component, and loss of control in lever pressing, following random administration or periods of reward interruption (Ishii et al. 2018; Koot et al. 2014; Richer et al. 2022). This could be related to the same neural changes described above. For example, administration of substances of abuse, i.e. cocaine or amphetamine, changes neural activity, producing an increase in the number of c-Fos positive cells in the striatum, PrL, Cg, Striatum and NAcc (Gill et al. 2014; Jenab et al. 2002). These same findings are present in animal models for the study of psychiatric diseases, such as schizophrenia. Drug-induced hyperactivity, such as Methylphenidate or amphetamines, alters the motor component producing hyperactivity, attention deficit, anxiety and social isolation (Monfil et al. 2018; Penner et al. 2002). It is therefore possible to hypothesize that this model shares similar behavioral and cerebral alterations found in Gambling, addiction, and mental disorders models (Molde et al. 2019; Pontes 2017; Turhan Gürbüz et al. 2021).

### Translational perspectives: comparison between the GD rats and GD patients

There are numerous studies of brain activity performed on GD patients. GD patients present changes in brain activity within different circuits involved in the control of different aspects of addiction. i.e., decision-making (Rivalan et al. 2011), mesocorticolimbic reward system (J.-T. Zhang et al. 2016), motor learning (Peters et al. 2017), and regions related to anxiety, stress, and emotional and motivated behaviors (Kirouac 2021). Although there are different types of analysis, our model induces GD in rats and demonstrates several similarities with GD patients.

The OFC, which plays an important role in maintaining and controlling attention (Menon and Uddin 2010), the PrL, involved in the cognitive control and regulation of the reward system (J.-T. Zhang et al. 2016), and the Cg, implicated in the desire induced by the game (Ko et al. 2013), are key regions of the decision-making process (Rivalan et al. 2011). Male patients with GD have increased functional connectivity in the resting state within OFC, PrL (Chen et al. 2016), and Cg (Ko et al. 2013), while nothing is reported about women with GD. In our GD rats, we observed alterations of c-Fos-ir in the same regions. In particular, we highlighted an enhancement of c-Fos-ir in both sexes in the OFC, while we observed opposite effects in the two sexes in the PrL and in the Cg, revealing an increased immunoreactivity in the males and a decrease in the females. The OFC also participates in the mesocorticolimbic reward system, receiving inputs from the Acb (Koob and Volkow 2010), which is required for the consolidation and mediation of the reward-gratifying effects of addictions (Koob and Volkow 2010). In GD male patients, the Acb exhibits a reduction in functional connectivity (Chen et al. 2016). However, in our model, male GD rats show an increase in c-Fos-ir, while we observed a diminution in the females.

Fundamental regions participating in the motor learning circuit are the VTA and SN (Peters et al. 2017), whose dopaminergic projections reach the PrL, the OFC, and the Acb, implied in the elaboration of reward and motivation (Volkow et al. 2017), and the lateral striatum (Peters et al. 2017), essential for conditioning processes and habit formation in addiction, which receives dopaminergic projections from the VTA and SN (Drui et al. 2014). The analysis of the functional connectivity in the resting state indicated a reduction of the activity within the VTA of the GD male patients, while an increase was observed in the Striatum and the SN (Wang et al. 2019). Our current data resembles these features. In fact, GD rats revealed a decrease in c-Fos-ir in the VTA and an increase in the SN in both sexes. As with GD patients, male GD rats also show an increase in neural activity in the striatum, while females show an opposite effect, emphasizing a possible sex difference in this region. However, dividing the striatum into lateral and medial, female GD rats continue to show a reduction in c-Fos-positive cells while males show an increase only in the lateral portion of the striatum. Lastly, the circuit involving the regions related to anxiety, stress, and emotional and motivated behaviors was analyzed, including the amygdala, linked to the processing and regulation of emotions (Kirouac 2021), the PVT, involved in the motivation, drug-seeking, and drug taking (Millan et al. 2017), and the BNST for its critical role in stress response and anxiety (Avery et al. 2016). In our model, c-Fos-ir was increased in the amygdala of both GD males and females, while in the PVT and in the BNST, it was increased in the males but decreased in the females, reflecting what happens in humans as far as PVT is concerned. Research performed in GD patients describes a reduction in the connectivity of those nuclei to other brain areas (Wang et al. 2022), except for the PVT, which displays greater activation among male, but not female, GD patients (Zhang et al. 2020). PVT is considered a key region that regulates approach/avoidance behavior in the emotional response (Choi and McNally 2017; Hsu et al. 2014; Kirouac 2015). Moreover, PVT, when the reward is omitted in an unpredictable way (as it happens in our experiment, specifically in Test 5), seems to modulate the expression of the exploratory foraging behavior, while if the result was expected, the PVT was not recruited (Do-Monte et al. 2017). The same sexually dimorphic expression of c-Fos in PVT (higher in male than female rats), observed in our GD group, was also found in another animal model of gambling, in which PVT was identified as a candidate region contributing to sex differences in risky decision making (Ishii et al. 2018).

### Limitations

GD is a complex psychiatric disease and animal models are able to capture specific endophenotypes of the disease, thus there are several limitations of our study: i. we looked at the main endophenotype of GD in brief period of time: loss of control (number of correct touches and game interaction) and hyperactivity (distance and speed traveled in the play zone).

Excessive use of video games in individuals with GD impairs social, work and financial relationships. In rodent models, the concept of “loss of essential aspects of life or loss of time” is limited to the time session, in which the activity is scheduled and does not limit social relationships at the end of the gaming session; ii. The long duration of the protocol is about 8 weeks, during which specific rooms in the animal facility have been used for much of the day; iii. high number of animals used. In fact, this model involves a large number of rats and some of them may be excluded in the selection before to start the GD protocol. In the tests with social stimulus, the number of animals is further increased, adding unfamiliar rats to the trained rats. iiii. The need of a dedicated experimental room for behavioral experiment and to purchase planned apparatus, camera fixed on the roof to register and touchscreen tablets.

## Conclusions

In the last decade, a wide range of epidemiological studies focused on neurobiological changes in GD patients (Casale et al. 2021; H.-Y. Wang and Cheng 2022). However, these studies are almost entirely performed in men since they are considered more susceptible to this disorder (Marraudino et al. 2022). Current clinical data is highly heterogeneous as a result of not uniform inclusion criteria (i.e., lack of demographic heterogeneity and equal representation of age and differences in the type of game and the follow-up periods) and of the various research methodologies applied (Kim et al. 2022). In this scenario, the new GD rat model, here described, could represent an innovative tool to investigate, in both sexes, the behavioral and neurobiological features of this disorder, the possible role of external factors, such as the social context, the predisposition and susceptibility, and the development of new pharmacological therapies.

## Electronic supplementary material

Below is the link to the electronic supplementary material.

Supplementary Material 1

Supplementary Material 2

Supplementary Material 3
