# Supplementary material for: Novel rat model of gaming disorder: assessment of social reward and sex differences in behavior and c-Fos brain activity
Source: Psychopharmacology (Berl). 2024 Apr 5;242(5):1103–22. doi: 10.1007/s00213-024-06576-y (PMC12043766; doi:10.1007/s00213-024-06576-y)
Supplement: Supplementary file 1 — Supplementary Material 1 [file 213_2024_6576_MOESM1_ESM.docx]

**Compliance with Ethical Standards**

**Disclosure of potential conflicts of interest**

All authors have no conflicts of interest to declare with respect to the research, authorship, and/or publication of this article.

**Animal Research**

Animal care and handling were according to the European Union Council Directive of 22nd September 2010 (2010/63/UE); all the procedures reported in the present study were approved by the Italian Ministry of Health (1035/2020-PR) and by the Ethical Committee of the University of Torino (Project n° 290132). The experimental design conforms to the ARRIVE guidelines originally published by Kilkenny et al. in 2010 (Kilkenny et al., 2010).
