# Supplementary material for: Novel rat model of gaming disorder: assessment of social reward and sex differences in behavior and c-Fos brain activity
Source: Psychopharmacology (Berl). 2024 Apr 5;242(5):1103–22. doi: 10.1007/s00213-024-06576-y (PMC12043766; doi:10.1007/s00213-024-06576-y)
Supplement: Supplementary file 3 — Supplementary Material 3 [file 213_2024_6576_MOESM3_ESM.docx]

**Novel rat model of Gaming Disorder: assessment of social reward and sex differences in behavior and c-Fos brain activity**

AUTHORS:

Antonino Casile^1,2,*,#^, Marilena Marraudino^2,3,*^, Brigitta Bonaldo^2,3,4^, Maria Vittoria Micioni Di Bonaventura^1^, Sofia Nasini^5^, Carlo Cifani^1,$#^ and Stefano Gotti^2,3,$^

^1^ University of Camerino, School of Pharmacy, Pharmacology Unit, Via Madonna delle Carceri, 9, 62032 Camerino (MC), Italy.

^2^ Neuroscience Institute Cavalieri Ottolenghi (NICO), Regione Gonzole, 10, Orbassano (TO), University of Turin, 10043 Turin, Italy.

^3^ Department of Neuroscience “Rita Levi-Montalcini”, Via Cherasco 15, 10126 Turin (TO), Italy.

^4^ Department of Health Sciences and Research Center on Autoimmune and Allergic Diseases (CAAD), University of Piemonte Orientale (UPO), Novara, Italy.

^5^ Laboratory of Molecular and Cellular Pharmacology, Department of Pharmacology, University of Padua, Largo Egidio Meneghetti, 2, 35131 Padua, Italy.

^*^ These authors equally contributed and should be considered as joint first authors.

^$^ These authors equally contributed and should be considered as joint last authors.

*^#^ Corresponding authors:*

*Antonino Casile*

*Work Address:* School of Pharmacy, University of Camerino, via Madonna delle Carceri 9, 62032

Camerino (MC), Italy

e-mail: antonino.casile@unicam.it

Phone: 0039 0737403302

*Carlo Cifani*

*Work Address:* School of Pharmacy, University of Camerino, via Madonna delle Carceri 9, 62032

Camerino (MC), Italy

e-mail: carlo.cifani@unicam.it

Phone: 0039 0737403302

**Supplementary Table 1.** ***Neuroanatomical regions analyzed for c-fos immunoreactivity****. The table reports the Bregma coordinates, the total number of levels, and the mean ROI area of each analyzed nucleus. ROI: region of interest.*

| **Nucleus** | **Bregma** | **N° of levels** | **Mean ROI area (µm^2^)** |
| --- | --- | --- | --- |
| **Cortex** | 2.70/-2.56 mm | 10/15 | 434560.725 |
| Prelimbic cortex (PrL) | 2.70/2.20 mm | 3 | 121406.056 |
| Primary motor cortex (M1) | 2.70/-2.30 mm | 10 / 12 | 45459.095 |
| Orbitofrontal cortex (OFC) | 2.70/-2.56 mm | 8/12 | 22571.449 |
| Cingulate cortex (Cg) | 1.60/0.70 mm | 4 | 61477.015 |
| **Nucleus Accumbens** | 2.70/0.70 mm | 6/8 | 54080.231 |
| Nucleus Accumbens core (AcbC) | 2.70/0.70 mm | 6/8 | 21035.513 |
| Nucleus Accumbens shell (AcbSh) | 1.20/0.70 mm | 3 | 33044.718 |
| **Striatum** | 1.60/0.70 mm | 3 | 19984.355 |
| Lateral striatum | 1.60/0.70 mm | 3 | 5645.325 |
| Medial striatum | 1.60/0.70 mm | 3 | 14339.030 |
| **Medial Septal Nucleus (MS)** | 1.60/0.70 mm | 4 | 31648.192 |
| **Bed nucleus of stria terminalis (BNST)** | - 0.92 mm | 1/3 | 38892.460 |
| **Lateral hypothalamic area (LH)** | - 0.92 mm | 1/3 | 19208.670 |
| **Supraoptic nucleus (SON)** | - 0.92 mm | 1/3 | 1147.705 |
| **Suprachiasmatic nucleus (SCh)** | - 0.92 mm | 1/3 | 844.809 |
| **Paraventricular thalamic nucleus (PVT)** | -1.80/-2.30 mm | 3/5 | 29641.310 |
| **Amygdala** | -1.88/-2.30 mm | 2/5 | 44761.803 |
| Central amygdaloid nucleus (CeM) | -1.88/-2.30 mm | 2/5 | 17322.898 |
| Basolateral amygdaloid nucleus (BLA) | -1.88/-2.30 mm | 2/5 | 18964.800 |
| Medial amygdaloid nucleus (Me) | -1.88/-2.30 mm | 2/5 | 8474.105 |
| **Ventral hippocampus** | -5.80/-6.04 mm | 2/3 | 164484.941 |
| **Ventral tegmental area (VTA)** | -5.60/-6.04 mm | 1/3 | 63042.950 |
| **Substantia nigra (SN)** | -5.60/-6.04 mm | 1/3 | 38121.337 |
| **Peripeduncular nucleus (PP)** | -5.60/-6.04 mm | 2 | 388.601 |
| **Dorsal raphe nucleus (DR)** | -8.00/-8.72 mm | 3 | 821.354 |
| **Median raphe nucleus (MnR)** | -8.00/-8.72 mm | 3 | 701.500 |
| **Pontine nucleus (Pn)** | -8.00/-8.72 mm | 3 | 678.587 |
| **Locus coeruleus (LC)** | -9.16/-9.75 mm | 3/4 | 340.410 |
| **Caudal Dorsal raphe nucleus (DRC)** | -9.16/-9.75 mm | 3/4 | 326.327 |

**Supplementary Table *2. Results obtained from the analysis of different behaviors within the Test phases.***

*Data are reported as Mean ± SEM. Two-way ANOVA (sex and treatment as independent variables) revealed a significant effect for p < 0.05 highlighted in bold.*

| **Test 1** | | | | | | |
| --- | --- | --- | --- | --- | --- | --- |
| **Parameters** | **CON-M** | **CON-F** | **GD-M** | **GD-F** | **ANOVA** | |
|  |  |  |  |  | ***F_(5.58)_*** | ***p*** |
| Frequency in play zone (N) | 12.580±0.67 | 13.380±0.89 | 11.67±0.81 | 11.67±0.81 | 0.95 | 0.424 |
| Cumulative duration in play zone (s) | 102.92±10.18 | 145.19±13.12 | 352.71±10.18 | 363.34±13.1  2 | 151.60 | **<0.001** |
| Latency to enter in Play zone (s) | 19-76±4.53 | 4.44±0.58 | 6.38±2.26 | 2.19±0.32 | 11.59 | **0.001** |
| Cumulative duration in the other zone (s) | 377.74±10.15 | 335.76±13.13 | 128.25±11.19 | 117.49±8.70 | 152.27 | **<0.001** |
| Distance traveled in play zone (cm) | 251.09±23.94 | 339.03±25.50 | 1238.34±49.21 | 1700.06±49.10 | 24.87 | **<0.001** |
| Distance traveled in the other zone (cm) | 1243.62±64.05 | 1095.43±72.61 | 667.24±61.00 | 700.24±91.92 | 97.41 | **<0.001** |
| Velocity in play zone (cm/s) | 2.49±0.18 | 2.41±0.15 | 3.52±0.15 | 4.74±0.30 | 23.04 | **<0.001** |
| Cumulative duration to game interaction (s) | 0.21±0.21 | 0.53±0.53 | 147.8±7.39 | 167.92±5.70 | 253.51 | **<0.001** |
| Correct touch (N) | 1.33±0.31 | 1.38±0.24 | 36.00±2.05 | 41.62±1.84 | 160.77 | **<0.001** |
| Protected rearing (N) | 37.25±2.96 | 34.15±3.35 | 14.78±1.50 | 16.48±1.62 | 26.16 | **<0.001** |
| Un- Protected rearing (N) | 8.25±1.94 | 1.38±0.69 | 1.44±0.32 | 1.10±0.35 | 14.63 | **<0.001** |
| Grooming (s) | 7.97±1.89 | 21.29±3.61 | 9.37±3.82 | 8.56±2.78 | 3.24 | **0.028** |
| **Adaptation to wheel** | | | | | | |
| **Parameters** | **CON-M** | **CON-F** | **GD-M** | **GD-F** | **ANOVA** | |
|  |  |  |  |  | ***F_(5.58)_*** | ***p*** |
| Frequency in play zone (N) | 8.17±1.01 | 6.46±0.72 | 8.68±0.58 | 10.55±0.78 | 4.88 | **0.004** |
| Cumulative duration in play zone (s) | 63.65±7.67 | 39.14±3.97 | 54.44±7.21 | 61.94±6.44 | 2.34 | **0.082** |
| Frequency in wheel zone (N) | 11.58±1.92 | 15.69±1.25 | 21.32±1.84 | 21.32±1.84 | 6.29 | **0.001** |
| Cumulative duration in wheel zone (s) | 88.87±19.81 | 149.65±13.69 | 135.64±12.17 | 135.64±12.17 | 2.86 | **0.044** |
| Distance traveled In Arena (cm) | 1692.81  ±83.45 | 1731.74±55.99 | 1771.29±50.78 | 2061.55±39.14 | 10.33 | **<0.001** |
| Distance traveled in play zone (cm) | 223.73±23.54 | 191.84±18.18 | 231.48±29.18 | 339.46±42.11 | 3.98 | **0.012** |
| Velocity in play zone (cm/s) | 3.75±0.31 | 4.80±0.36 | 4.84±0.28 | 5.54±0.26 | 5.61 | **0.002** |
| Cumulative duration to game interaction (s) | 0.77±0.77 | 0.14±0.14 | 7.88±3.59 | 12.54±3.40 | 3.79 | **0.015** |
| Correct touch (N) | 0.00±0.00 | 0.00±0.00 | 0.00±0.00 | 0.00±0.00 | 1.48 | 0.230 |
| Grooming (s) | 25.66±16.91 | 25.66±3.10 | 5.07±0.83 | 3.93±3.32 | 2.06 | 0.114 |
| Cumulative duration to exploration wheel (s) | 237.79±17.77 | 331.67±9.53 | 351.69±1.45 | 313.48±0.83 | 14.40 | **<0.001** |
| Protected g rearing (N) | 26.92±2.34 | 20.31±1.81 | 12.63±1.67 | 25.60±2.02 | 11.67 | **<0.001** |
| Un-Protected rearing (N) | 0.92±0.34 | 0.62±0.27 | 0.68±0.30 | 1.20±0.42 | 0.60 | 0.617 |
| **Test 2** | | | | | | |
| **Parameters** | **CON-M** | **CON-F** | **GD-M** | **GD-F** | **ANOVA** | |
|  |  |  |  |  | ***F_(5.58)_*** | ***p*** |
| Frequency in play zone (N) | 8.17±0.59 | 6.38±0.89 | 7.63±0.69 | 10.71±0.59 | 7.47 | **<0.0001** |
| Cumulative duration in play zone (s) | 45.80±5.31 | 38.38±7.28 | 121.55±15.72 | 245.01±12.57 | 59.05 | **<0.0001** |
| Frequency in wheel zone (N) | 18.42±1.64 | 17.23±1.49 | 18.42±1.79 | 17.23±1.77 | 3.54 | **0.020** |
| Cumulative duration in wheel zone (s) | 175.82±  14.410 | 232.65±27.69 | 125.38±1.77 | 76.37±11.31 | 17.96 | **<0.0001** |
| Distance traveled In Arena (cm) | 1712.82±103.13 | 1596.69±120.72 | 1710.85±65.11 | 2376.82±105.93 | 14.61 | **<0.0001** |
| Distance traveled in play zone (cm) | 174.82±20.48 | 162.79±31.67 | 514.74±79.97 | 1064.19±74.32 | 39.68 | **<0.0001** |
| Velocity in play zone (cm/s) | 3.87±0.32 | 4.25±0.37 | 3.72±0.16 | 4.44±0.29 | 1.58 | 0.202 |
| Cumulative duration to game interaction (s) | 0.00±0.00 | 0.00±0.00 | 16.90±10.65 | 62.31±0.86 | 22.54 | **<0.0001** |
| Correct touch (N) | 1.00±.0.30 | 1.23±0.77 | 18.68±16.27 | 113.09±28.04 | 156.24 | **<0.0001** |
| Grooming (s) | 13.65±3.11 | 23.88±9.41 | 10.41±6.12 | 12.60±8.08 | 2.90 | **0. 042** |
| Cumulative duration to exploration wheel (s) | 285.05±12.08 | 313.74±14.92 | 286.00±18.24 | 154.18±8.46 | 29.28 | **<0.0001** |
| Protected rearing (N) | 24.00±1.51 | 19.31±2.92 | 11.00±0.99 | 12.10±1.29 | 12.83 | **<0.0001** |
| Un-Protected g rearing (N) | 0.75±0.37 | 0.31±0.17 | 0.57±1.36 | 0.46±1.03 | 12.83 | 0. 548 |
| **Test 3** | | | | | | |
| **Parameters** | **CON-M** | **CON-F** | **GD-M** | **GD-F** | **ANOVA** | |
|  |  |  |  |  | ***F_(5.58)_*** | ***p*** |
| Frequency in play zone (N) | 5.92±1.07 | 6.46±1.16 | 6.00±0.90 | 14.40±0.94 | 19.54 | **<0.0001** |
| Cumulative duration in play zone (s) | 21.20±3.20 | 42.120±13.39 | 59.60±12.5  9 | 291.70±14.23 | 108.09 | **<0.0001** |
| Latency to enter in Play zone (s) | 86.28±37.77 | 116.86±41.05 | 30.61±8.88 | 20.72±8.88 | 3.91 | **0.013** |
| Frequency in Soci-exual zone (N) | 19.83±1.62 | 13.38±1.76 | 17.61±1.27 | 16.45±1.30 | 2.76 | 0.050 |
| Cumulative duration in Socio-sexual zone (s) | 367.24±21.78 | 328.97±23.02 | 335.21±22.02 | 154.72±11.90 | 27.78 | **<0.0001** |
| Distance traveled In Arena (cm) | 1257.92±  92.83 | 1069.54±  132.65 | 1184.29±81.44 | 2891.92±98.65 | 12.48 | **<0.0001** |
| Distance traveled in play zone (cm) | 82.51±12.81 | 136.49±27.42 | 251.32±49.85 | 1703.87±92.44 | 153.49 | **<0.0001** |
| Velocity in play zone (cm/s) | 3.87±0.22 | 3.73±0.15 | 4.94±0.31 | 5.79±0.25 | 12.48 | **<0.0001** |
| Cumulative duration to game interaction (s) | 0.09±0.09 | 0.72±0.72 | 23.92±3.70 | 125.22±5.79 | 215.39 | **<0.0001** |
| Correct touch (N) | 0.50±0.19 | 0.54±0.39 | 7.17±1.40 | 39.65±2.10 | 157.80 | **<0.0001** |
| Grooming (s) | 62.78±2.15 | 24.67±4.76 | 56.64±7.80 | 16.87±2.15 | 14.17 | **<0.0001** |
| Protected rearing (N) | 19.75±0.08 | 19.31±0.10 | 9.50±6.05 | 18.40±7.50 | 7.81 | **<0.0001** |
| Un-Protected rearing (N) | 0.08±1.50 | 0.15±2.51 | 0.17±0.12 | 0.60±0.17 | 1.71 | 0.175 |
| Cumulative duration to socio-sexual interaction (s) | 215.79±14.19 | 185.21±21.71 | 173.05±1.43 | 81.83±1.68 | 23.42 | **<0.0001** |
| **Test 4** | | | | | | |
| **Parameters** | **CON-M** | **CON-F** | **GD-M** | **GD-F** | **ANOVA** | |
|  |  |  |  |  | ***F_(5.58)_*** | ***p*** |
| Frequency in play zone (N) | 5.50±0.75 | 7.31±1.00 | 10.33±0.97 | 13.38±0.84 | 14.10 | **<0.0001** |
| Cumulative duration in play zone (s) | 25.11±4.92 | 45.94±8.58 | 173.82±21.24 | 348.73±13.48 | 97.73 | **<0.0001** |
| Latency to enter in Play zone (s) | 100.47±41.93 | 48.81±13.61 | 27.11±7.78 | 9.13±2.53 | 4.81 | **0.005** |
| Frequency in Social zone (N) | 14.33±1.53 | 12.54±1.12 | 14.33±0.82 | 11.19±0.54 | 2.98 | **0.038** |
| Cumulative duration in Social zone (s) | 373.13±29.64 | 347.46±24.24 | 292.80±25.43 | 122.53±11.78 | 28.83 | **<0.0001** |
| Distance traveled In Arena (cm) | 1142.02±129.80 | 1167.84±113.78 | 1930.33±149.22 | 3088.67±115.50 | 53.79 | **<0.0001** |
| Distance traveled in play zone (cm) | 104.31±15.93 | 166.26±30.33 | 902.81±132.18 | 2189.43±105.06 | 95.59 | **<0.0001** |
| Velocity in play zone (cm/s) | 3.87±0.32 | 3.70±0.28 | 4.94±0.19 | 6.31±0.25 | 24.79 | **<0.0001** |
| Cumulative duration to game interaction (s) | 0.65±0.62 | 0.20±0.20 | 73.73±10.12 | 152.96±8.0 | 91.07 | **<0.0001** |
| Correct touch (N) | 0.08±0.08 | 0.15±0.10 | 23.00±3.05 | 41.19±1.87 | 90.32 | **<0.0001** |
| Grooming (s) | 5.17±6.41 | 2.38±4.12 | 5.39±4.73 | 8.14±3.70 | 2.72 | **0.034** |
| Protected rearing (N) | 15.33±1.88 | 20.46±2.64 | 13.06±1.68 | 18.33±0.90 | 3.64 | **0.018** |
| Un-Protected rearing (N) | 0.08±0.08 | 0.15±  0.10 | 0.50±3.05 | 1.05±1.87 | 2.82 | **0.046** |
| Cumulative duration to Social interaction (s) | 241.96±13.01 | 185.97±19.82 | 152.23±11.37 | 67.82±6.55 | 39.27 | **<0.0001** |
| **Test 5** | | | | | | |
| **Parameters** | **CON-M** | **CON-F** | **GD-M** | **GD-F** | **ANOVA** | |
|  |  |  |  |  | ***F_(x.y)_*** | ***p*** |
| Frequency in play zone (N) | 12.58±0.67 | 13.38±0.89 | 12.63±0.50 | 14.00±0.97 | 0.64 | **0.590** |
| Cumulative duration in play zone (s) | 102.92±10.18 | 150.43±12.12 | 303.87±11.91 | 349.87±6.90 | 136.70 | **<0.0001** |
| Latency to enter in Play zone (s) | 18.19±4.79 | 4.48±0.48 | 5.40±0.81 | 3.06±0.46 | 13.12 | **<0.0001** |
| Cumulative duration in the other zone (s) | 377.74±10.15 | 335.76±13.12 | 237.21±11.90 | 162.16±8.93 | 78.36 | **<0.0001** |
| Distance traveled In Arena (cm) | 1142.02±105.98 | 1167.84±113.78 | 1930.33±149.22 | 3020.32±104.56 | 55.82 | **<0.0001** |
| Distance traveled in play zone (cm) | 127.57±21.99 | 263.71±114.34 | 902.81±132.18 | 2152.90±99.38 | 78.00 | **<0.0001** |
| Velocity in play zone (cm/s) | 3.91±0.89 | 3.68±0.99 | 4.94±0.19 | 6.30±0.21 | 28.32 | **<0.0001** |
| Cumulative duration to game interaction (s | 0.23±0.21 | 0.00±0.00 | 188.42±10.57 | 201.26±8.96 | 150.56 | **<0.0001** |
| Correct touch (N) | 1.42±0.29 | 2.683±1.37 | 43.61±2.38 | 51.81±2.70 | 108.27 | **<0.0001** |
| Grooming (s) | 7.35±1.78 | 20.88± | 9.87±1.78 | 7.23±1.78 | 7.56 | **<0.0001** |
| Protected rearing (N) | 36.50±2.87 | 34.50±3.59 | 25.33±1.78 | 23.04±2.43 | 5.79 | **<0.0001** |
| Un-Protected rearing (N) | 7.67±2.87 | 1.50±3.33 | 1.78±2.17 | 1.48±2.43 | 11.34 | **<0.0001** |

**Supplementary Table *3. Results obtained from the analysis of c-Fos-ir in all selected nuclei.*** *Data (number of c-Fos positive cells) are reported as Mean ± SEM. Two-way ANOVA (sex and treatment as independent variables) or nested ANOVA (when there are multiple sections per animal) revealed a significant effect for p < 0.05, highlighted in bold. OFC = orbitofrontal cortex; PrL= prelimbic cortex; Cg = cingulate cortex; Acb = nucleus accumbens; M1 = primary motor cortex; VTA = ventral tegmental area; Striatum; PVT = periventricular nucleus of the thalamus; BNST = bed nucleus of stria terminalis; MS = medial septal nucleus; LH = lateral hypothalamic area; SON = supraoptic nucleus; SCh = suprachiasmatic nucleus; SN = substantia nigra; PP = peripeducular nucleus ; DR = dorsal raphe nucleus; MnR = median raphe nucleus; Pn = pontine nucleus. LC = locus coeruleus; DRC = causal dorsal raphe nucleus.*

| **Nucleus** | **Bregma**  **(mm)** | **Comparable levels** | **CON M** | **CON F** | **GD M** | **GD F** | **ANOVA** | |
| --- | --- | --- | --- | --- | --- | --- | --- | --- |
|  |  |  |  |  |  |  | ***F_(3.16)_*** | ***p*** |
| **Decision-making circuits** | | | | | | | | |
| **OFC** | 2.20 | 1 | 1179.86±146.73 | 556.97±43.28 | 2151.75±102.42 | 1604.30±136.55 | 34.59 | **<0.001** |
| **PrL** | 2.20 | 1 | 2748.20±370.48 | 1435.20±110.71 | 4387.20±297.98 | 5935.80±1003.24 | 12.30 | **<0.001** |
| **Cg** | 0.70 | 1 | 462.00±56.76 | 622.00±87.65 | 1330.00±100.10 | 335.25±27.911 | 32.44 | **<0.001** |
| **Mesocorticolimbic Reward System** | | | | | | | | |
| **OFC** | 2.20 | 1 | 1179.86±146.73 | 556.97±43.28 | 2151.75±102.42 | 1604.30±136.55 | 34.59 | **<0.001** |
| **Acb** | 1.00 | 1 | 777,98±64,37 | 877,97±54,72 | 1246,38±85,81 | 401,75±37,23 | 30.36 | **<0.001** |
| AcbC | 1.00 | 1 | 409,3333±73,69 | 393,3438±80.33 | 644,0000±63,08 | 140,6250±35,57 | 9.87 | **0.001** |
| AcbSh | 1.00 | 1 | 276,75±45,14 | 484,63±36,24 | 602,38±35,67 | 261,13±41,14 | 17,40 | **<0.001** |
| **Motor learning circuit** | | | | | | | | |
| **M1** | 2.20 | 1 | 1495.60±42.50 | 1458.70±184.15 | 3141.30±135.17 | 1859.93±75.91 | 49.12 | **<0.001** |
| **VTA** | 5.60/-6.04 | 3 | 456.71±14.59 | 256.06±26.10 | 330.46±29.05 | 108.73±10.68 | 28.10 | **<0.001** |
| **Striatum** | 1.00 | 1 | 779.00±44.03 | 1157.25±108.15 | 1184.25±121.43 | 527.88±64.18 | 18.70 | **<0.001** |
| Lateral | 1.00 | 1 | 356.38±18.43 | 536.88±57.53 | 604.75±77.72 | 191.50±17.07 | 28.68 | **<0.001** |
| Medial | 1.00 | 1 | 422.63±40.35 | 620.38±56.26 | 579.50±45.53 | 336.38±57.44 | 11.13 | **0.003** |
| **Emotional and motivated behaviors** | | | | | | | | |
| **PVT** | 2.30 | 3 | 97.12±20.86 | 349.12±23.96 | 188.50±14.56 | 52.75±4.85 | 98.08 | **<0.001** |
| **BNST** | 0.92 | 3 | 182.76±20.89 | 274.80±25.14 | 343.76±19.60 | 101.96±10.86 | 56.37 | **<0.001** |
| **Amygdala** | 2.30 | 3 | 885.17±61.71 | 260.33±26.89 | 1235.08±43.50 | 1037.96±66.52 | 34.33 | **<0.001** |
| CeA | 2.30 | 3 | 336,60±24,60 | 88,80±10,41 | 475,20±22,78 | 403,60±44,09 | 49.43 | **<0.001** |
| BLA | 2.30 | 3 | 391,40±28,21 | 114,20±7,41 | 546,00±44,28 | 446,00±26,46 | 26.21 | **<0.001** |
| MeA | 2.30 | 3 | 139,20±9,32 | 56,60±9,79 | 215,40±9,24 | 188,60±23,54 | 18.01 | **<0.001** |
| **Other brain regions** | | | | | | | | |
| **Cortex** | 1.88/-2.56 | 5 | 18596.62±1113.98 | 9708.20±834.90 | 23593.00±1488.38 | 19440.00±1570.84 | 33.01 | **<0.001** |
| **MS** | 1.00 | 1 | 316.75±30.56 | 290.75±32.01 | 568.00±66.35 | 126.00±10.48 | 20.58 | **<0.001** |
| **LH** | 0.92 | 3 | 240.06±11.64 | 259.70±14.22 | 172.10±15.44 | 188.16±21.71 | 9.17 | **0.004** |
| **SON** | 0.92 | 3 | 26.73±1.84 | 36.06±0.80 | 29.96±3.37 | 73.86±5.97 | 25.42 | **0.002** |
| **SCh** | 0.92 | 1 | 41.10±4.93 | 65.60±7.256 | 73.80±8.379 | 110.20±12.49 | 10.80 | **<0.001** |
| **Ventral hippocampus** | 5.80 | 2 | 1193.91±159.33 | 1190.78±87.24 | 1854.250±87.00 | 643.2±71.70 | 38.40 | **0.002** |
| **SN** | 5.60/-6.04 | 3 | 232.92±26.16 | 99.77±18.48 | 335.433±26.93 | 247.13±24.88 | 33.73 | **<0.001** |
| **PP** | 5.60 | 1 | 112.95±7.46 | 104.70±4.26 | 65.30±3.36 | 48.70±1.35 | 43.81 | **<0.001** |
| **DR** | 8.72 | 1 | 236.60±206.94 | 457.26±384.52 | 348.20±323.75 | 28.60±14.42 | 148.29 | **<0.001** |
| **MnR** | 8.72 | 1 | 190.60±12.09 | 284.00±19.85 | 254.00±11.41 | 16.60±3.80 | 83.944 | **<0.001** |
| **Pn** | 8.72 | 1 | 340.00±49.99 | 438.90±38.84 | 545.30±38.81 | 269.00±22.14 | 9.630 | **0.001** |
| **LC** | 9.30 | 1 | 84.10±9.03 | 94.90±10.23 | 60.10±7.47 | 13.10±1.74 | 21.53 | **<0.001** |
| **DRC** | 9.16 | 1 | 43.20±11.40 | 54.80±7.50 | 93.00±5.67 | 9.60±1.80 | 21.33 | **<0.001** |
